# A revision of the genus *Protorthodes* McDunnough with descriptions of a new genus and four new species (Lepidoptera, Noctuidae, Noctuinae, Eriopygini)

**DOI:** 10.3897/zookeys.421.6664

**Published:** 2014-06-27

**Authors:** J. Donald Lafontaine, J. Bruce Walsh, Clifford D. Ferris

**Affiliations:** 1 Canadian National Collection of Insects, Arachnids, and Nematodes, Biodiversity Program, Agriculture and Agri-Food Canada, KW Neatby Bldg., C.E.F., Ottawa, Ontario, Canada K1A 0C6; 2 Dept of Ecology and Evolutionary Biology, Biosciences West, University of Arizona Tucson, AZ USA 85721; Research Associate: McGuire Center for Lepidoptera and Biodiversity, Florida Museum of Natural History, University of Florida, Gainesville, Florida, USA; 3 5405 Bill Nye Ave., R.R. 3, Laramie, Wyoming 82070, USA. Research Associate: McGuire Center for Lepidoptera and Biodiversity, Florida Museum of Natural History, University of Florida, Gainesville, Florida, USA; C. P. Gillette Museum of Arthropod Diversity, Colorado State University, Fort Collins, Colorado,USA

**Keywords:** Taxonomy, Noctuinae, Eriopygini, *Protorthodes*, *Nudorthodes*, Arizona, California, Texas, Mexico

## Abstract

The genus *Protorthodes* McDunnough is revised to include 15 species including *P. ustulata* Lafontaine, Walsh & Ferris, **sp. n.**, from southwestern United States, *P. texicana* Lafontaine, **sp. n.**, from Texas and Mexico, and *P. mexicana* Lafontaine, **sp. n.**, from Mexico. A new genus, *Nudorthodes* Lafontaine, Walsh & Ferris, is proposed for two species formerly included in *Protorthodes*, *P. texana* (Smith, 1900), **comb. n.**, and *P. variabilis* (Barnes & McDunnough, 1912), **comb. n.**, and *N. molino* Lafontaine, Walsh & Ferris, **sp. n.**, described from southern Arizona. A key to species, descriptions, illustrations of adults and genitalia, and distribution maps are included.

## Introduction

The genus *Protorthodes* McDunnough was proposed in 1943 for a group of 17 species formerly included in *Orthodes* Guenée. The genus was later expanded to include 21 species (Franclemont and Todd 1983), and then reduced to 15 species by Lafontaine and Schmidt (2010) through new synonymies with *Protorthodes incincta* (Morrison). Here we illustrate and diagnose the 12 known species of *Protorthodes*, describe three new ones, and move two of them to a new genus, *Nudorthodes* Lafontaine, Walsh & Ferris, and we describe a new species of *Nudorthodes*.

## Materials and methods

### Repository abbreviations

Specimens were examined from the following collections:

AMNH American Museum of Natural History, New York, New York, USA

AWCC Arizona Western College Collection, Yuma, Arizona, USA

BMNH The Natural History Museum [statutorily, British Museum (Natural History)], London, UK

CDF Personal collection of Clifford D. Ferris, Laramie, Wyoming, USA

CNC Canadian National Collection of Insects, Arachnids, and Nematodes, Ottawa, Ontario, Canada

CUIC Cornell University Insect Collection, Ithaca, New York, USA

FMNH The Field Museum, Chicago, Illinois, USA

IAW Personal collection of Ian A. Watkinson, Yuma, Arizona, USA

JBW Personal collection of J. Bruce Walsh, Tucson, Arizona, USA

TLSRC Texas Lepidoptera Survey Research Collection, Edward C. Knudson, Houston, Texas, USA

USNM National Museum of Natural History (formerly, United States National Museum), Washington, District of Columbia, USA

**Dissecting methods and terminology.** Dissection of genitalia and terms for genital structures and wing markings follow Lafontaine (2004).

**Barcode sequences and analysis.** Barcodes sequences, 658 base-pair sequences of the mitochondrial cytochrome oxidase *c* subunit (CO1 barcodes), were obtained from the Barcode of Life Data (BOLD) System at the University of Guelph, Ontario, Canada, through collaboration with Paul D. N. Hebert. The barcode sequences were compared using neighbour-joining trees constructed using the Kimura-2-Parameter distance model as provided on the BOLD Systems website (www.boldsystems.org) (Ratnasingham and Hebert 2007).

## Systematics

### Protorthodes

Taxon classificationAnimaliaLepidopteraNoctuidae

McDunnough, 1943

#### Type species.

*Taeniocampa curtica* Smith, 1890, by original designation.

#### Diagnosis.

**Adults.** Males and females of similar size (forewing length 11–17 mm). Vestiture of palpi, head, and thorax of long apically-forked or apically-serrated scales, sometimes with a slightly-raised central tuft near front of thorax. *Head* – Labial palpus porrect, apical segment about ½ as long as second segment. Frons rounded, covered with strap-like scales projecting over it from sides and top. Eye covered with surface hair. Male antenna biserrate (like toothed edge of a saw) to bipectinate (lateral processes mainly parallel sided like a feather) with lateral processes 0.5–4.0 × as long as central shaft. Female antenna filiform, setose ventrally. *Thorax* – *Wings*: Forewing ground color typically gray, brown, or orange; pattern variable, typically with reniform and orbicular spots with a pale or black outline, usually lower part or all of reniform spot filled with gray, which is darker than the ground color; postmedial line dentate or with inner element straight, outer element broken into series of dots on veins; subterminal line pale and sinuate in most species with dark wedges or shading along inner margin. Hindwing white to fuscous. *Legs*: Tibiae without spiniform setae. Tarsal segments 1–4 with three ventral rows of spiniform setae, and four ventral rows on tarsal segment 5. *Abdomen* – Base of abdomen without basal abdominal brushes. Eighth abdominal sternum of male with tuft of long setae on a short eversible coremata. *Male genitalia* – Uncus typically slender, slightly swollen mesially, tapered at apex to hook-like process. Valve broadest beyond middle, tapered to slight “neck” defining apical cucullus; sacculus more heavily sclerotized dorsally than ventrally with dorsal part crenulate and setose in some species; clasper a sclerotized plate in middle of valve distal to sacculus from which arises a long, heavily-sclerotized ampulla projecting posterodorsally, extending almost to, or beyond, dorsal margin of valve; ampulla centrally swollen in most species; digitus arising from large sclerotized plate in middle of valve, tapered posterolaterally into heavily-sclerotized pointed or blunt process projecting below ventral margin of valve at neck of cucullus; cucullus covered with long inward-projecting setae and with no defined apical corona. Vesica usually twisted or coiled above base with numerous pouches or diverticula basally and subbasally; a long, heavily-sclerotized basal or subbasal cornutus in most species; vesica 1–2 × as long as aedeagus. *Female genitalia* – Corpus bursae thin and membranous, rounded or oval, without obvious signa. Appendix bursae typically with one or two short coils. Ductus bursae variably sclerotized, usually about as long as corpus bursae. Abdominal segment eight about 2 × as long as wide; anterior apophyses 0.5–2.0 × as long as abdominal segment eight; posterior apophyses folding near middle, about 2.5 × as long as anterior apophyses. Ovipositor telescoping and projecting well beyond end of abdomen in most specimens. Anal papillae long and tapered, 0.5–1.0 × as long as abdominal segment eight; anal papillae lightly sclerotized, setae mainly confined to apical area.

#### Note.

Adults of *Protorthodes* are most likely to be confused with those of *Homorthodes* McDunnough and *Nudorthodes* (described below), but the male antennae in *Protorthodes* species are biserrate to bipectinate, whereas those of *Homorthodes* and *Nudorthodes* are filiform, without lateral processes.

#### Larva and habits.

Most species are associated with relatively xeric habitats, but not open deserts, preferring open, dry shrubby or forested areas, especially with pines or fir. The larvae hide in the leaf litter during the day and feed at night on a variety of herbs and low-growing shrubs. The species overwinter as partly grown larvae and emerge as adults between spring and autumn. Adults are nocturnal. The larva can be associated with the tribe Eriopygini by the lack of teeth on the ridges on the inner surface of the mandible, in combination with the hypopharynx lacking a transverse groove that divides the hypopharynx in related tribes into anterior and posterior lobes. Within the Eriopygini, *Protorthodes* is unique in having a pale transverse area on the posterior part of the prothoracic shield, and in having a pair of sclerotized plates between the bases of the abdominal prolegs; the genus also is characterized by the pavement-granulose integument, setae arising from pinacula, head mainly dark with pale patches forming a reticulate pattern, spinneret 2 × as long as the basal segment of the labial palpus, the apical seta of the labial palpus (Lp-2) is less than ½ as long as the basal segment of the palpus (Lp-1), and the spines on the hypopharynx are similar throughout, without the proximal-lateral row of short stout spines found in many other genera. A key to species based on larvae is in Crumb (1956) and Godfrey (1972).

#### Key to species of *Protorthodes* and *Nudorthodes* (Adults)

**Table:** 

1	Eye surface hairy (dissecting scope or strong magnifying lens needed); male antenna biserrate to bipectinate; vesica in male genitalia 1–2 × as long as aedeagus; anal papillae mainly smooth, setose only apically (*Protorthodes*)	2
–	Eye surface smooth, hairless; male antenna filiform; vesica in male genitalia 4–5 × as long as aedeagus; anal papillae setose throughout (*Nudorthodes*)	30
2	Male	3
–	Female	17
3	Male antenna bipectinate; pectinations (at least on anterior side) longer that width of antennal shaft and parallel sided	4
–	Male antenna biserrate; serrations no longer that width of antennal shaft and tapered to a point	13
4	Anterior antennal pectinations about 1.5 × as long as shaft width	5
–	Anterior antennal pectinations 2–5 × as long as shaft width	8
5	Hindwing pearly white basally with increasing fuscous shading distally and on veins; forewing orbicular spot rounded, defined by black outline; vesica without a basal cornutus or diverticula; mainly southwestern (Texas to California and Mexico), and farther north in western US to southern Oregon	*Protorthodes alfkenii*
–	Hindwing pale fuscous basally, darker fuscous distally, forewing orbicular spot usually obscure or absent; vesica with large basal cornutus and several diverticula	6
6	Forewing dark gray brown with fine black streaks; maculation obscure except for black spot in lower part of reniform spot and a series of minute black wedges defining straight subterminal line; western Great Plains (Alberta to Colorado) and Great Basin (Idaho to Utah, Nevada, eastern California)	*Protorthodes eureka*
–	Forewing varying shades of gray, brown, and reddish brown; subterminal line pale, sometimes with diffuse dark wedges on inner margin of line	7
7	Forewing gray brown, buffy brown, or reddish brown; subterminal line sinuate near middle; clasper of male with small swollen area immediately above base; widespread in open xeric habitats in Great Plains and southwestern US and locally in Great Basin and Great Lakes States	*Protorthodes incincta*
–	Forewing rusty brown; subterminal line straight or slightly curved; clasper of male with elongated swollen area centered on basal third; widespread in dry forested areas from British Columbia to southern California, extending eastward to western Montana and Ruby Mountains of Nevada	*Protorthodes curtica*
8	Anterior pectinations (rami) of male antenna 4 × as long as central shaft; reniform spot on forewing inconspicuous except for three or four white spots on outer margin; digitus long and tapered; Arizona	*Protorthodes antennata*
–	Anterior pectinations of male antenna 2 × as long as central shaft; reniform spot on forewing darker than ground color and surrounded by pale outline; digitus short and swollen subapically	9
9	Male hindwing white with some fuscous shading on wing margin and veins; basal part of vesica with swollen lobes projecting to each side; western Texas to southern Utah and California	*Protorthodes melanopis*
–	Male hindwing fuscous; basal part of vesica cylindrical	10
10	Dorsal margin of valve with elongated spine-covered process extending to base of clasper; Edwards Plateau of west-central Texas and Mexico	11
–	Dorsal margin of valve without spiny process	12
11	Forewing brown or gray brown; hindwing fuscous on outer half; clasper with ventral process tapered to blunt point; Edwards Plateau of west-central Texas to southern Mexico (Chiapas)	*Protorthodes texicana*
–	Forewing pale whitish gray; hindwing white; clasper with ventral process broad and spatulate apically; known only from Guadalajara, Mexico	*Protorthodes mexicana*
12	Forewing reddish brown or purplish brown; reniform spot with rounded edges, kidney shaped; coil in vesica near base; occurring across Canada and northern United States southward in the East to northern Florida and in the West in the Rocky Mountain region to Colorado and Utah	*Protorthodes oviduca*
–	Forewing gray brown with dusting of white scales and white-lined maculation giving wing a frosted appearance; reniform spot parallel sided, rectangular; coil in vesica near middle; eastern Texas	*Protorthodes orobia*
13	Forewing with black basal dash and black streak distal to reniform spot; hindwing white with dark terminal line; male genitalia with clasper swollen mesially; New Mexico, east-central Arizona	*Protorthodes argentoppida*
–	Forewing without a basal dash or dark streak distal to reniform spot; hindwing fuscous; male genitalia with clasper even in width	14
14	Forewing orange with transverse lines prominent; reniform spot with lower half very large and filled with dark-gray shading; sacculus lightly sclerotized; western Texas to southeastern Arizona and southern Mexico	*Protorthodes mulina*
–	Forewing variable; transverse lines usually obscure; reniform spot kidney shaped, lower part not larger; dorso-posterior part of sacculus heavily sclerotized and spiculate	15
15	Forewing ground color burnt orange, darker on costa with fine white crosslines; valve long, narrow subapically; digitus lightly sclerotized and not extending ventral to lower margin of valve; Colorado, New Mexico, Arizona, western Texas	*Protorthodes ustulata*
–	Forewing variable; costal area not particularly darker and without fine white crosslines on costa; valve broad, expanded subapically; digitus heavily sclerotized and extending below ventral margin of valve	16
16	Forewing reddish brown, orange brown, or pale gray brown; digitus projecting posteroventrally below ventral margin of valve; Washington to southwestern California	*Protorthodes rufula*
–	Forewing pale gray brown; digitus projecting anteroventrally below ventral margin of valve; western Texas to south-central California and central Mexico	*Protorthodes perforata*
17	Forewing with black basal dash and black streak distal to reniform spot; New Mexico, eastern Arizona	*Protorthodes argentoppida*
–	Forewing without a basal dash or dark streak distal to reniform spot	18
18	Forewing orange with transverse lines prominent; reniform spot with lower half very large and filled with dark-gray shading; western Texas to southeastern Arizona and southern Mexico	*Protorthodes mulina*
–	Forewing variable; transverse lines usually obscure; reniform spot kidney shaped, lower part not larger	19
19	Ductus bursae with enlarged sclerotized area anterior to ostium	20
–	Ductus bursae cylindrical or tapered anteriorly from ostium	24
20	Reniform and orbicular spots defined by pale outline	21
–	Outline of reniform and orbicular spots with dark and light elements	22
21	Anterior 2/3 of ductus bursae with longitudinal sclerotized ridges; Washington to southwestern California	*Protorthodes rufula*
–	Anterior part of ductus bursae membranous; western Texas to south-central California and central Mexico	*Protorthodes perforata*
22	Forewing dark gray brown with fine black streaks; maculation obscure except for black spot in lower part of reniform spot and a series of minute black wedges defining straight subterminal line; western Great Plains (Alberta to Colorado) and Great Basin (Idaho to Utah, Nevada, eastern California)	*Protorthodes eureka*
–	Forewing varying shades of gray, brown, and reddish brown; subterminal line pale, sometimes with diffuse dark wedges on inner margin of line	23
23	Forewing gray brown, buffy brown, or reddish brown; subterminal line sinuate near middle; widespread in open xeric habitats in Great Plains and southwestern US and locally in Great Basin and Great Lakes States	*Protorthodes incincta*
–	Forewing rusty brown; subterminal line straight or slightly curved; widespread in dry forested areas from British Columbia to southern California, extending eastward in northern US to western Montana and Ruby Mountains of Nevada	*Protorthodes curtica*
24	Ductus bursae with sclerotized plate on each side projecting posteriorly over ostium	25
–	Ductus bursae without specialized plates projecting posteriorly	28
25	Sclerotized plates fused centrally to form a double-lobed plate over ventral part of ostium; west-central Texas to southern Mexico (Chiapas) [female of *Protorthodes mexicana* unknown; would probably key out here]	*Protorthodes texicana*
–	Sclerotized plates on each side of ductus bursae with middle part of ductus membranous	26
26	Forewing light gray brown; central part of ductus bursae with smooth heavily-sclerotized plate; western Texas to southern Utah and California	*Protorthodes melanopis*
–	Forewing brown to reddish brown; central part of ductus bursae with lightly-sclerotized transversely-striated plate	27
27	Forewing reddish brown or purplish brown; reniform spot with rounded edges, kidney shaped; eastern North America west to Texas, Utah, and British Columbia	*Protorthodes oviduca*
–	Forewing gray brown with dusting of white scales and white-lined maculation giving wing a frosted appearance; reniform spot parallel sided, rectangular; Gulf Coast area of Texas	*Protorthodes orobia*
28	Ductus bursae mainly membranous with a sclerotized ring at posterior end; Arizona	*Protorthodes antennata*
–	Posterior ¾ of ductus bursae heavily sclerotized	29
29	Forewing with reniform and orbicular spots outlined in white; hindwing fuscous; Colorado, New Mexico, Arizona, western Texas	*Protorthodes ustulata*
–	Forewing with reniform and orbicular spots outlined in black; hindwing white basally with increasing fuscous shading distally and on veins; mainly southwestern (Texas to California and northern Mexico), farther north in western US to southern Oregon	*Protorthodes alfkenii*
30	Forewing with medial line forming broad dark area from reniform spot to postmedial line; male genitalia with digitus broad and foot-like apically; southeastern Arizona	*Nudorthodes molino*
–	Forewing with medial line, if present, a thin line extending down from reniform spot; male genitalia with digitus narrow and tapered apically	31
31	Forewing with orbicular spot pale and contrasting, outlined in black; male genitalia with dorsal process of sacculus almost square, so posterior margin of process straight or slightly curved; Texas to California northward to Oregon and Nevada	*Nudorthodes texana*
–	Forewing with transverse lines usually sharply defined in black against a dull-gray or gray-brown ground color; medial line present; male genitalia with posterior margin of dorsal process of sacculus with posteriorly-directed lobe toward top of process; southwestern California	*Nudorthodes variabilis*

### Species accounts

#### Protorthodes curtica

Taxon classificationAnimaliaLepidopteraNoctuidae

(Smith, 1890)

**Figures 1–18.**
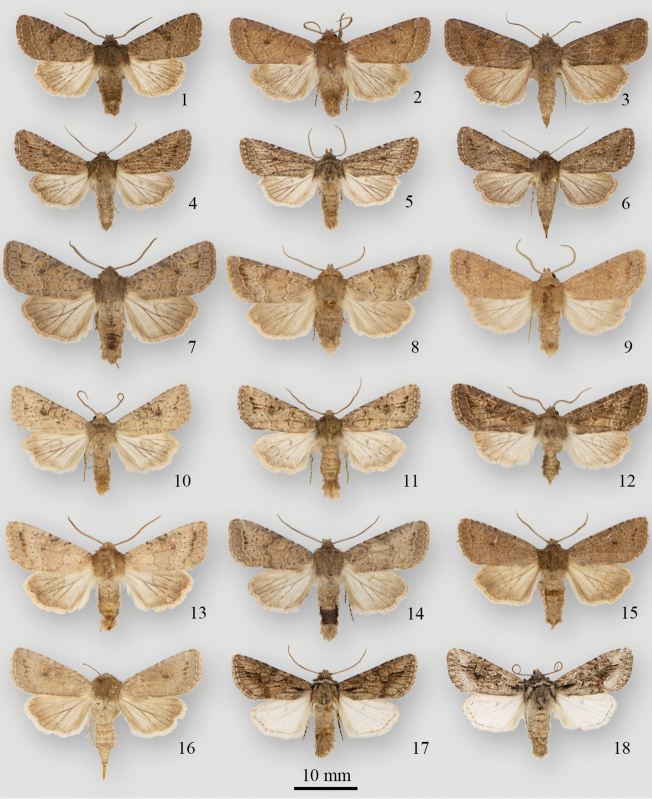
*Protorthodes* adults **1** *Protorthodes curtica* ♂, USA, California, San Diego Co., Palomar Mountain Rd, 4800’ **2** *Protorthodes curtica* ♂, USA, Washington, Douglas Co., Pine Canyon 4 mi ENE Orono, 2200–2360’ **3** *Protorthodes curtica* ♀, Canada, British Columbia, Seton Lake **4** *Protorthodes eureka* ♂, USA, Colorado, Moffat Co., Dinosaur National Monument, Harper’s Corner Road Canyon Overlook, 7840’ **5** *Protorthodes eureka* ♂, USA, Wyoming, 28 mi NW Riverton 5700’ **6** *Protorthodes eureka* ♀, USA, Colorado, Moffat Co., Dinosaur National Monument, Harper’s Corner Road Canyon Overlook, 7840’ **7** *Protorthodes incincta* ♂, USA, Wyoming, Laramie 7500’, 41°17'N, 105°31'W **8** *Protorthodes incincta* ♂, Canada, Alberta, Lethbridge **9** *Protorthodes incincta* ♂, Canada, Alberta, Manyberries, Dominion Range Station **10** *Protorthodes incincta* ♂, USA, Colorado, Moffat Co., Dinosaur National Monument, Poole Creek Canyon 5200’ **11** *Protorthodes incincta* ♂, USA, Wyoming, 7498’ (2287 m), 41°17.866'N, 105°31.519'W **12** *Protorthodes incincta* ♂, USA, Colorado, Chaffee Co., Buena Vista 7800’ **13** *Protorthodes incincta* ♂, Canada, Alberta, Dinosaur Prov. Park 2100’, 50°41'N, 111°30'W **14** *Protorthodes incincta* ♂, USA, New Mexico, Grant Co., 4360’, 32°50.86’N, 108°35.56 W **15** *Protorthodes incincta* ♂, Canada, Ontario, Long Point, 42°34.82’N, 80°24.61 W **16** *Protorthodes incincta* ♀, USA, Nevada, 7 mi S Silver Springs, 4200’ 1**7** *Protorthodes argentoppida* ♂, USA, New Mexico, Sandoval Co., La Jara Canyon, Jemez Mts, 7400’ **18** *Protorthodes argentoppida* ♂, USA, New Mexico, Catron Co., 6200’, 33°39.99'N, 108°52.34 W.

**Maps 1–6.**
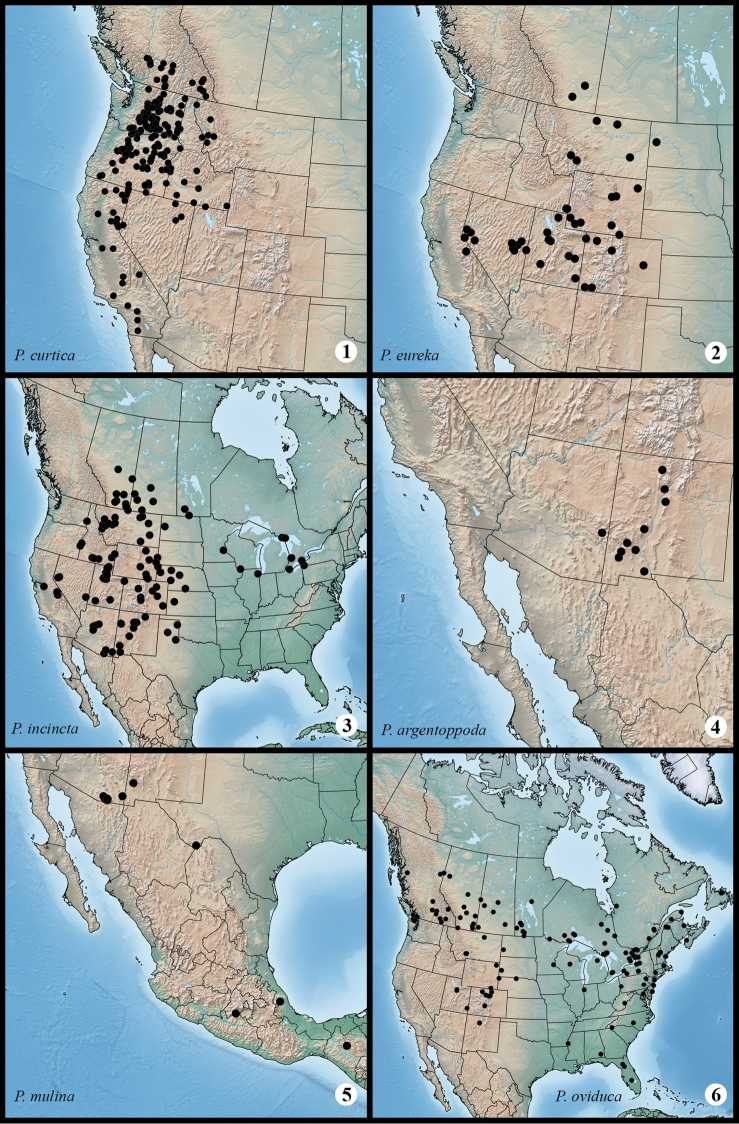
Distribution of examined material for *Protorthodes* species. **1** *Protorthodes curtica* **2** *Protorthodes eureka* **3** *Protorthodes incincta* **4** *Protorthodes argentoppida* **5** *Protorthodes mulina* **6** *Protorthodes oviduca*.

Figs 1–3 , 55 , 73 ; Map 1

Taeniocampa curtica Smith, 1890: 122.
Taeniocampa bostura Smith, 1908b: 103.

##### Type material.

*Taeniocampa curtica*: lectotype ♂, examined, AMNH. Type locality: USA, California, Sierra Nevada, designated by Todd (1982). *Taeniocampa bostura*: holotype ♂, examined, AMNH. Type locality: Canada, British Columbia, Kaslo.

##### Diagnosis.

*Protorthodes curtica* is the dominant species of *Protorthodes* in the Pacific Northwest, especially in the intermontane region between the Cascades and the Rocky Mountains. Superficially, adults usually can be identified by the dark-reddish tint on the forewing ground color, the pale, even-curved subterminal line that follows the wing margin, the even band of dark shading along the inner edge of the subterminal line, and the reniform is faintly outlined by a pale line. Forewing length varies from 12 to 16 mm. The male antenna of *Protorthodes curtica* is narrowly bipectinate, similar to those of *Protorthodes incincta*, the species with which *Protorthodes curtica* is most likely to be confused. The antenna of *Protorthodes curtica* is not as wide with the maximum width of the antenna being 2.6–2.9 × as wide as the central shaft, whereas it is 3.7–3.9 × as wide in *Protorthodes incincta*. The range of *Protorthodes curtica* is mainly to the west of the range of *Protorthodes incincta*. Populations of *Protorthodes incincta* where its range overlaps that of *Protorthodes curtica* usually have gray-brown or buffy-brown forewings with an irregular pale subterminal line that is margined on the inner side with dark shading that usually forms a series of larger wedges in the area distal to the reniform spot. The reniform spot of *Protorthodes curtica* is outlined with a dark line with a pale line inside this, and the forewing apex is not as acutely pointed as in *Protorthodes curtica*. The male and female genitalia of *Protorthodes curtica* and *Protorthodes incincta* are similar, but the digitus of *Protorthodes curtica* tends to be pointed where it projects beyond the ventral margin of the valve, whereas in *Protorthodes incincta* the apex of the digitus is tapered to a point.

##### Distribution and biology.

*Protorthodes curtica* occurs from the interior of southern British Columbia southward in the West Coast states, mainly to the east of the Cascades and Coastal Ranges, to southern California. It occurs in the Rocky Mountains in Idaho and Montana and in the Ruby Mountains of Nevada. Adults occur from late June until mid-October, mainly in dry forested habitats. The larva was described by Crumb (1956) and Godfrey (1972).

#### Protorthodes eureka

Taxon classificationAnimaliaLepidopteraNoctuidae

(Barnes & Benjamin, 1927)

Figs 4–6 , 56 , 74 ; Map 2

Eriopyga eureka Barnes & Benjamin, 1927: 6.

##### Type material.

*Eriopyga eureka*: holotype ♂, examined, USNM. Type locality: USA, Utah, Eureka.

##### Diagnosis.

Adults of *Protorthodes eureka* can be recognized by relatively small size (forewing length: 11–13 mm), narrow forewings, and longitudinally-streaked pattern resulting from the dark-colored veins and lighter-brown color between the veins. A series of black sagittate spots is on the inner side of the almost straight subterminal line. The male antenna and male and female genitalia of *Protorthodes eureka* are similar to those of *Protorthodes curtica*, but the right clasper of *Protorthodes eureka* extends beyond the dorsal margin of the valve by about 1/3 of the length of the clasper, whereas in *Protorthodes curtica* it exceeds the dorsal margin of the valve by less than 1/5 of its length.

##### Distribution and biology.

*Protorthodes eureka* occurs from southern Alberta southward in the western Great Plains to Colorado and in the Great Basin to east-central California and southwestern Colorado. Adults occur mainly in open xeric, especially sagebrush prairie and open pinyon-juniper woodlands. Adults occur from early August until late September. The larva was described by Crumb (1956) and Godfrey (1972).

#### Protorthodes incincta

Taxon classificationAnimaliaLepidopteraNoctuidae

(Morrison, 1874)

Figs 7–16 , 57 , 75 ; Map 3

Mamestra incincta Morrison, 1874: 156.
Taeniocampa utahensis Smith, [1888]: 473.
Orthodes akalus Strecker, 1899: 6.
Agrotis saturnus Strecker, 1900: 31.
Graphiphora communis race *smithii* Dyar, 1904: 868.
Taeniocampa indra Smith, 1906: 233.
Eriopyga melanopis var. *coloradensis* Strand, [1917]: 29, syn. rev.
Eriopyga daviesi Barnes & Benjamin, 1927: 5.

##### Type material.

*Mamestra incincta*: 3 syntypes. No type material of this species was reported from MSU by Wilterding (1997), so the male syntype in USNM, examined, may be the only extant syntype. Type locality: Illinois. *Taeniocampa utahensis*: lectotype ♂, USNM, designated by Todd (1982), examined. Type locality: USA. Utah. *Orthodes akalus*: holotype ♀, FMNH, photograph examined. Type locality: USA, Colorado. *Agrotis saturnus*: holotype ♂ FMNH, photograph examined. Type locality: USA, southern Wisconsin. *Graphiphora communis* race *smithii*: holotype ♀, USNM, examined. Type locality: USA, Illinois. Note – the type locality for this taxon was given as “Canada, British Columbia, Kootnai District” by Poole (1989). However, the taxon was proposed for a singleton female from Illinois that Smith confused with the species Dyar described from British Columbia, therefore Dyar proposed the racial name *smithii* for this unique specimen. *Taeniocampa indra*: lectotype ♂, AMNH, designated by Todd (1982), examined. Type locality: USA, Arizona, Yavapai County, Minnehaha. *Eriopyga daviesi*: holotype ♂, USNM, examined. Type locality: USA, New Mexico, Fort Wingate. *Orthodes melanopis* var. *coloradensis*: holotype ♂, BMNH, examined. Type locality: SW Colorado. Hampson (1905: 299) listed this specimen as “*Eriopyga melanopis* ab. 1.” Strand ([1917]: 29) proposed the name *Eriopyga melanopis* var. *coloradensis* for it. Poole (1989), assuming the name wasproposed as an aberration, as Strand usually did, credited McDunnough (1938: 74) as validating the name by using it as a subspecies of *Orthodes melanopis*. However, the specimen is herein reidentified as *Protorthodes incincta*, not *Protorthodes melanopis*, so the name is hereby transferred to the synonymy of *Protorthodes incincta*.

##### Diagnosis.

*Protorthodes incincta* is so variable in appearance that it is almost easier to identify it be eliminating the other species. The ground color varies from pale whitish gray, through various shades of brown, orange, and gray to blackish gray. Forewing length varies from 11–14 mm. The moths are most likely to be confused with those of *Protorthodes curtica* that mainly occurs father to the west. *Protorthodes incincta* can be distinguished by the more irregular pale subterminal line, concentration of dark sagittate marks proximal to the subterminal line to the area distal to the reniform spot, and other characters of maculation, antenna, and male genitalia listed under *Protorthodes curtica*. The palest forms (e.g., Fig. 16) occur in xeric areas of Nevada, Arizona and New Mexico and previously were known as *Protorthodes indra*, and the most contrastingly marked forms (e.g., Figs 11, 12) are localized in southern Wyoming, Colorado, and northern New Mexico and previously were known as *Protorthodes daviesi*. However, both forms blend into more typical forms in these areas and do not differ in structural characters or barcodes from other populations.

##### Distribution and biology.

*Protorthodes incincta* is mainly a species of the western Great Plains and dry open forests of the Rocky Mountain region, with range extensions into the Great Basin, the American Southwest, and eastward in relict prairie areas into the Great Lakes region. Adults occur from early June until early October. The larva was described by Crumb (1956) and Godfrey (1972).

#### Protorthodes argentoppida

Taxon classificationAnimaliaLepidopteraNoctuidae

McDunnough, 1943

Figs 17 , 18 , 58 , 76 ; Map 4

Protorthodes argentoppida McDunnough, 1943: 52.

##### Type material.

*Protorthodes argentoppida*: holotype ♂ CNC, examined. Type locality: USA, New Mexico, Silver City.

##### Diagnosis.

The name of this species is derived from the silvery-gray ground color of the forewing. This, in combination with the prominent black basal dash on the forewing and the white somewhat translucent hindwing, make this the easiest species of *Protorthodes* to identify. In some males there is a narrow black terminal line and broken postmedial line on the hindwing and in the female the hindwing has a pale smoky tint to the white color. Forewing length varies from 13 to 16 mm. The male antenna is biserrate, unlike other species in this group, with the maximum with of the antenna being 1.9–2.1 × as wide as the central shaft. The male and female genitalia are similar to those of the other three species in the *Protorthodes incincta* group (*Protorthodes curtica*, *Protorthodes eureka*, *Protorthodes incincta*), surprising, because of the divergent external appearance of the moth. However, the cucullus is smaller, the two digiti apically truncated and more symmetrical, the subbasal diverticula in the vesica are smaller, and the basal cornutus larger than in the other three species in the group.

##### Distribution and biology.

*Protorthodes argentoppida* has a very limited range, occurring in xeric forested areas of various mountain ranges in New Mexico and in the White Mountains in east-central Arizona. Adults occur from mid-May until early July. The immature stages are unknown.

#### Protorthodes mulina

Taxon classificationAnimaliaLepidopteraNoctuidae

(Schaus, 1894)

**Figures 19–36.**
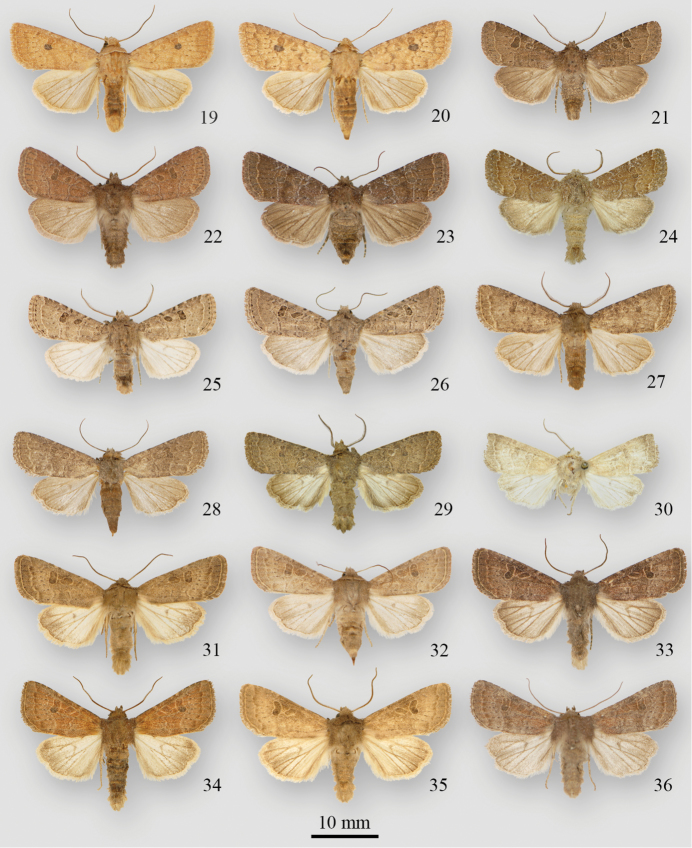
*Protorthodes* adults **19** *Protorthodes mulina* ♂, USA, Arizona, Cochise Co., Huachuca Mts, Ash Canyon, 5100’ **20** *Protorthodes mulina* ♀, USA, Arizona, Cochise Co., Huachuca Mts, Ash Canyon, 5100’ **21** *Protorthodes oviduca* ♂, USA, Florida, Marion Co., W. Anthony Rd, 1.4 mi WSW Anthony **22** *Protorthodes oviduca* ♂, USA, Colorado, Grand Co., Hot Sulphur Springs, 7670’ **23** *Protorthodes oviduca* ♀, USA, Florida, Marion Co., W. Anthony Rd, 1.4 mi WSW Anthony **24** *Protorthodes orobia* ♂, USA, Texas, San Patricio Co., Sinton **25** *Protorthodes melanopis* ♂, USA, New Mexico, Grant Co., Mangas Springs **26** *Protorthodes melanopis* ♀, USA, New Mexico, Eddy Co., Carlsbad Caverns National Park **27** *Protorthodes texicana* ♂ holotype, USA, Texas, Uvalde Co., Concan, 1300’ **28** *Protorthodes texicana* ♀ paratype, USA, Texas, Uvalde Co., Concan **29** *Protorthodes texicana* ♂ paratype, Mexico, Chiapas, San Cristóbal de las Casas, 7200’ **30** *Protorthodes mexicana* ♂ holotype, Mexico, Jalapa, Guadalahara **31** *Protorthodes perforata* ♂, USA, California, San Diego Co., Jacumba, 2850’ **32** *Protorthodes perforata* ♀, USA, California, San Diego Co., Scissor’s Crossing **33** *Protorthodes rufula* ♂, USA, Oregon, Josephine Co., Mi 6-13 Illinois River Road **34** *Protorthodes rufula* ♂, USA, California, San Diego Co., Torrey Pines State Reserve **35** *Protorthodes rufula* ♂, USA, California, Sonoma Co., Petaluma **36** *Protorthodes rufula* ♂, USA, California, San Diego Co., Mira Mesa at Penasquitos Canyon.

Figs 19 , 20 , 59 , 77 ; Map 5

Taeniocampa mulina Schaus, 1894: 237.
Hyssia pseudochroma Dyar, 1913: 288.

##### Type material.

*Taeniocampa mulina*: syntypes in USNM, examined. Type locality: Mexico, [Veracruz], Jalapa. *Hyssia pseudochroma*: holotype ♂, USNM, examined. Type locality: Mexico, [Veracruz], Zacualpan.

##### Diagnosis.

*Protorthodes mulina* is easily recognized by the orange or yellow-orange ground color of the forewing with the maculation defined by darker orange-brown lines, and especially by the enlarged lower lobe of the reniform spot that is filled with a dark blue gray. Forewing length varying from 13 to 17 mm. The male and female genitalia are divergent from other species in the genus, although several features suggest a relationship to the *Protorthodes incincta* group; the vesica has a basal cornutus, the subbasal diverticula in the vesica are similar to those of species in the *Protorthodes incincta* group, but in *Protorthodes mulina* these structures are much larger than those of other species in the *Protorthodes incincta* group and the vesica is about twice as long. Unique features of the male genitalia are the enlarged, rounded cucullus, wider than the valve, unlike other species of *Protorthodes*, the clasper is almost straight with an abrupt 90°bend near the apex, and the digitus is reduced, lying entirely along the inner surface of the valve with the apex slightly expanded and forked. The female genitalia, like other species in the *Protorthodes incincta* group, has an amorphous sclerotized mass on the posterior part of the ductus bursae; the position and shape of the lobes of the corpus bursae are unique.

##### Distribution and biology.

*Protorthodes mulina* undoubtedly has a wide range in Mexico, occurring as far south as the state of Chiapas, but is known from very few localities. In the United States it occurs from western Texas to southeastern Arizona. Adults are found in May and June and again from mid-August to early November, probably representing two generations. The larva was described by Crumb (1956) and Godfrey (1972).

#### Protorthodes oviduca

Taxon classificationAnimaliaLepidopteraNoctuidae

(Guenée, 1852)

Figs 21–23 , 60 , 78 ; Map 6

Taeniocampa oviduca Guenée, 1852: 357.
Taeniocampa capsella Grote, 1874b: 201.
Protorthodes lindrothi Krogerus, 1954: 20.

##### Type material.

*Taeniocampa oviduca*: type material lost. Type locality: North America. Note: no species is likely to be confused with *Taeniocampa oviduca* in the areas of eastern North America from where Guenée’s material originated, mainly the Southeast (Georgia and Florida), and the Northeast (mainly New York and eastern Canada), so no neotype is proposed. *Taeniocampa capsella*: syntype ♂, BMNH, examined. Type locality: New York, Albany. *Protorthodes lindrothi*: holotype ♂, CNC, examined. Type locality: Canada, Newfoundland, Badger.

##### Diagnosis.

*Protorthodes oviduca* is mainly a boreal-zone species occurring in Canada from coast to coast. Because of its mainly northern and eastern distribution, its range overlaps that of only two other species, *Protorthodes curtica* in British Columbia and *Protorthodes incincta* in southern Canada and the Rocky Mountain region. Adults can be recognized by the reddish-brown coloration of the forewing and the contrastingly pale outline of the reniform and orbicular spots with the reniform spot usually entirely filled with dark shading. The male antenna is strongly bipectinate, 3.8 to 4.1 × as wide as the central shaft. The forewing length varies from 11 to 14 mm. This and the next two species (*Protorthodes orobia* and *Protorthodes melanopis*) form a structurally similar species group in which the ampulla of the clasper is relatively short and teardrop shaped, and does not extend to the dorsal margin of the valve; the digitus is a broad triangular sclerotized plate extending from the costal margin of the valve and tapers ventrally into heavily-sclerotized process with the apex covered with short spines and minute setae and ends near the middle of the valve, not near the “neck” of the cucullus as in other species. The female genitalia of the group are characterized by the sclerotized area toward the posterior end of the ductus bursae, which is heavily sclerotized on each side of the ductus with the central area being only lightly sclerotized. *Protorthodes oviduca* can be distinguished from the other two species in the group by the characters given in the key.

##### Distribution and biology.

*Protorthodes oviduca* occurs across boreal and temperate areas of Canada and northern United States with extensions in eastern US to central Florida and southern Alabama, and in the mountains in the West as far south as Colorado and Utah. In some areas (e.g., Ohio, Michigan) it is found only in sandy habitats (Eric Metzler, pers. comm.). Adults occur mainly from mid-May to early July with occasional records as late as mid-August. The larva was described by Crumb (1956) and Godfrey (1972).

#### Protorthodes orobia

Taxon classificationAnimaliaLepidopteraNoctuidae

(Harvey, 1876)

**Maps 7–12.**
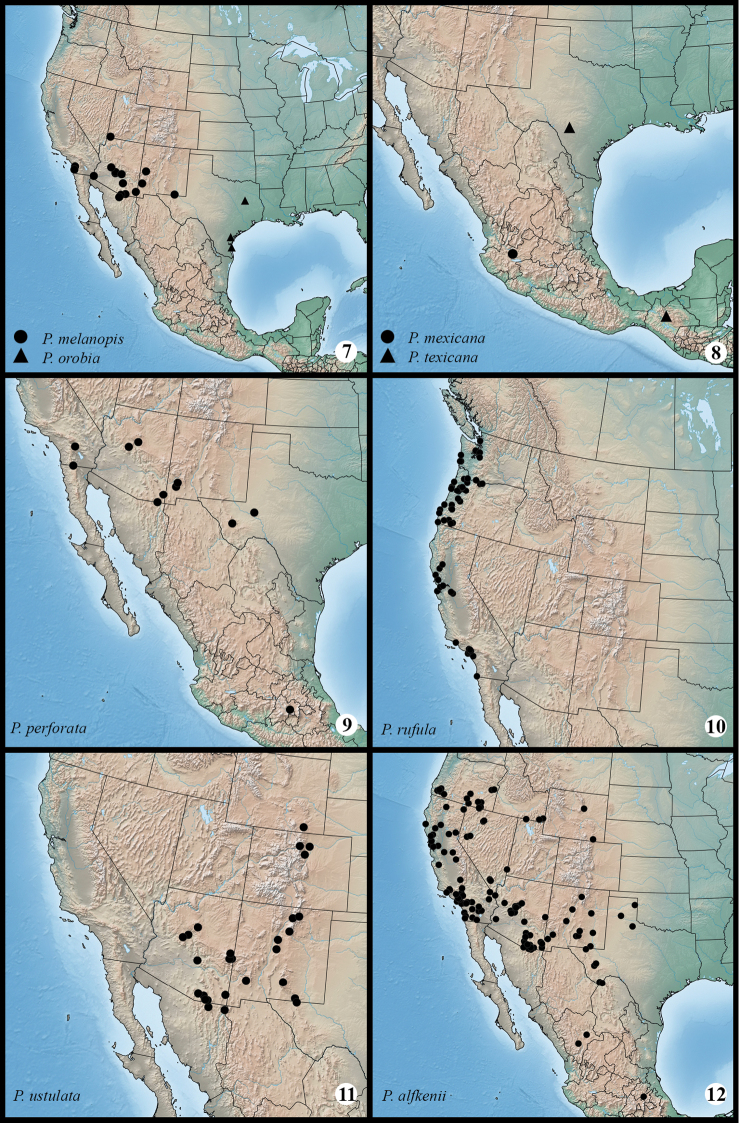
Distribution of examined material for *Protorthodes* species. **7** *Protorthodes orobia* and *Protorthodes melanopis* **8** *Protorthodes texicana* and *Protorthodes mexicana* **9** *Protorthodes perforata* **10** *Protorthodes rufula* **11** *Protorthodes ustulata* 12 *Protorthodes alfkenii*.

Fig. 24 , 61 , 79 ; Map 7

Mamestra orobia Harvey, 1876: 154.

##### Type material.

*Mamestra orobia*: syntype ♂, BMNH, examined. Type locality: Texas.

##### Diagnosis.

*Protorthodes orobia* is closely related to *Protorthodes oviduca*, but can be recognized by the gray-brown color of the forewing with a dusting of white scales that gives the wing a hoary appearance; the maculation is defined by thin white lines with the transverse lines represented on the costa by seven wider white spots that immediately separate this species from *Protorthodes oviduca*. Unlike *Protorthodes oviduca*, the reniform and orbicular spots of *Protorthodes orobia* are concolorous with the rest of the forewing, being defined only by the white outline. The white subterminal line of *Protorthodes orobia* is white and contrasting, not yellow buff with red shading on its inner margin as in *Protorthodes oviduca*. Structurally, the two species differ by the characters of the vesica given in the key.

##### Distribution and biology.

*Protorthodes orobia* is known only from eastern Texas with most records being on the Gulf Coast. All adults were collected in October. The immature stages are unknown.

#### Protorthodes melanopis

Taxon classificationAnimaliaLepidopteraNoctuidae

(Hampson, 1905)

Figs 25 , 26 , 62 , 80 ; Map 7

Eriopyga melanopis Hampson, 1905: 299.

##### Type material.

*Eriopyga melanopis*: syntypes 4 ♂, 1 ♀, BMNH, examined. Type locality: Arizona, Maricopa Co., Phoenix.

##### Diagnosis.

*Protorthodes melanopis* is most easily recognized by the pale gray-brown forewing ground color, which emphasizes the contrast between the reniform spot, orbicular spot, and the ground color. The pale hindwings of the males contrast with the forewings. Forewing length varies from 11 to 14 mm. In males the hindwing is white and translucent, with some fuscous shading on the veins and wing margin; in females the hindwing is covered with a fuscous sheen, darker on the veins and wing margin. The antennal pectinations and wing size are the same as in *Protorthodes oviduca*. Structurally, the species differs from *Protorthodes oviduca* by the vesica character given in the key. The coil in the vesica in *Protorthodes melanopis* is mesial rather than basal, and the dorsal lobe on the sacculus tapers to a pointed process, whereas the dorsal lobe of the sacculus is rounded in *Protorthodes oviduca* and *Protorthodes orobia*.

##### Distribution and biology.

*Protorthodes melanopis* occurs across the southern United States from western Texas to southern California. Its range extends as far north as southern Utah and as far south as northern Mexico. Adults were collected from late February to early May and again from mid-August to late September. The larva was described by Godfrey (1972).

#### Protorthodes texicana

Taxon classificationAnimaliaLepidopteraNoctuidae

Lafontaine sp. n.

http://zoobank.org/AB0E914C-193A-483D-9561-170B352D9062

Figs 27–29 , 63 , 81 ; Map 8

##### Type material.

**Holotype** ♂. USA, Texas, Uvalde Co., Concan, Neals Lodges, 1300’, at uv, 3 April 1990, Noel McFarland. CNC. **Paratypes:** 22 ♂, 23 ♀. Same locality and collector as for holotype, 23 March 1990 (1 ♀); USA, Texas, Uvalde Co., Concan, 3 Oct. 1992, E. Knudson (1 ♂). Mexico, Chiapas, San Cristóbal de las Casas, 7200’, 4–29 May 1969, J.E.H. Martin (21 ♂, 22 ♀.). Paratypes deposited in CNC, TLSRC, USNM.

##### Etymology.

This species is both Texan and Mexican, thus the name *texicana*.

##### Diagnosis.

*Protorthodes texicana* can be recognized by the brown forewings with the transverse lines defined by pale buff and dark-brown lines, and the reniform and orbicular spots darker brown than the ground color with a pale buff outline. *Protorthodes texicana* is most likely to be confused with *Protorthodes oviduca* or *Protorthodes orobia*, however, *Protorthodes texicana* but does not have the reddish tints to the forewing of *Protorthodes oviduca*, and has by the less contrasting white lines on the forewing than *Protorthodes orobia*. The male genitalia of *Protorthodes texicana* are characterized by large, heavily-sclerotized, spinulose hood-like structure that projects posteriorly from the dorsal margin of the sacculus, and the clasper-digitus combination that creates a structure with three processes; the homologies of this assemblage are best interpreted by comparison with the more rudimentary form found in *Protorthodes oviduca*. The female genitalia are characterized by the large, heavily-sclerotized, double-lobed plate covering the ostium bursae ventrally.

##### Description.

**Adult.** Male and female similar in size, color, and maculation. Forewing length: 12–14 mm. **Head** – Male antenna with individual segments bipectinate, 3.8–4.0 × as wide as the central shaft (anterior rami 1.9–2.1 × as wide, posterior rami 1.4–1.5 × as wide). Female antenna filiform, minutely setose ventrally. Palpi and head clothed with spatulate apically-forked setae, pale buff or gray basally, dark-brown patch subapically, with pale-gray and pale buff tips creating a brown color with a hoary overlay. **Thorax** – Covered with similar scales to those of head; with slightly raised prothoracic and metathoracic tufts. *Legs*: Appearing speckled with mixture of buff and darker gray-brown scales. Tibiae without spiniform setae. Tarsi with three ventral rows of spiniform setae. *Wings*: Dorsal forewing pale brown with dusting of darker-brown scales; subbasal, antemedial, postmedial, and subterminal lines buff, partially bordered by darker-brown scales; reniform spot gray-brown, darker than forewing and with pale-buff outline, with slight constriction on anterior and posterior margin, giving it a figure 8 shape; orbicular spot similar in color, rounded or obliquely oval; fringe with two irregular rows of spatulate scales, pale-brown at base, darker toward tips. Dorsal hindwing pale fuscous basally with darker fuscous on discal spot, wing veins, and marginal area of wing; fringe pale fuscous white with irregular darker fuscous medial line. **Male genitalia** – Uncus mainly evenly tapered from base to apex except slightly swollen laterally subbasally, sparely covered with long hair-like setae, apex compressed and flattened. Valve gradually tapered from base, abruptly constricted subapically to define a rounded cucullus; sacculus sclerotized, about 0.4 × length of valve, dorsal margin extended into elongated, hollow, hood-like process with outer surface covered by spines, its apex extending to base of ampulla of clasper; ventral base of clasper extending from sacculus ending in rounded lobe from which arises a heavily-sclerotized, partially articulated, tear-drop-shaped process projecting posteriorly; dorsal base of clasper articulates with long heavily-sclerotized ampulla of clasper with apex spatulate, extending posterodorsally beyond costal margin of valve; digitus appearing to be a heavily-sclerotized extension from editum; apex of digitus a stout apically blunt process with short conical setae-tipped spines at apex; cucullus densely covered with long, stiff setae projecting anterodorsally, and no apical corona; central part of transtilla densely spinulose; juxta a long narrow plate, broad basally, tapered posteriorly with subapical constriction. Aedeagus extended ventrally into short spine-like tip free of vesica. Vesica about 2 × as long as aedeagus; vesica with submedial coil and ½ postmedial coil, the latter with short diverticulum on posterior side; vesica without spines or cornuti. **Female genitalia** – Corpus bursae membranous, rounded, without signa. Appendix bursae with two short coils, arising from posterior dorsal surface of corpus bursae. Ductus bursae 1.5 × as long as corpus bursae, anterior 2/3 membranous, posterior 1/3 covered with thin, longitudinal sclerotized striations; a heavily sclerotized, double-lobed plate covering ostial part of ductus bursae and anterior-ventral part of abdominal segment eight; abdominal segment eight 1.5 × as long as wide, lightly sclerotized; anterior apophyses 0.5 × as long as abdominal segment eight; posterior apohyses folding near middle, about 2.5 × longer than anterior apophyses. Anal papillae long and tapered, 0.5 × as long as abdominal segment eight; anal papillae lightly sclerotized, long setae sparsely scattered over surface, short setae abundant near apex of papillae.

##### Distribution and biology.

The immature stages are unknown. *Protorthodes texicana* is known from two areas, west-central Texas and southern Mexico. Adults were collected between late March and late May and in early October. The immature stages are unknown.

#### Protorthodes mexicana

Taxon classificationAnimaliaLepidopteraNoctuidae

Lafontaine sp. n.

http://zoobank.org/C80FBEEF-2BA2-4C96-966C-7517C3CD38EA

Figs 30 , 64 ; Map 8

##### Type material.

**Holotype** ♂. Mexico, Jalapa, Guadalahara, 28–30 April 1961, Howden & Martin, CNC.

##### Etymology.

This species is known only from Mexico, thus the species name *mexicana*.

##### Diagnosis.

*Protorthodes mexicana* is closely related to *Protorthodes texicana*. *Protorthodes mexicana* can be recognized by the pale whitish-buff color of the forewing, the white translucent hindwing. In the male genitalia the ventral process of the clasper assemblage is apically spatulate in *Protorthodes mexicana* (apically tapered in *Protorthodes texicana*), and the vesica has two full coils (1½ in *Protorthodes texicana*).

##### Description.

**Adult male.** (Female unknown). Forewing length: 12 mm. **Head** – Male antenna with individual segments bipectinate, 3.8 × as wide as the central shaft (anterior rami 1.9 × as wide, posterior rami 1.4 × as wide). Palpi and head clothed with spatulate apically-forked setae, pale whitish, some with pale-brown patch subapically, with whitish-buff tips. **Thorax** – Covered with similar scales to those of head; with slightly raised prothoracic and metathoracic tufts. *Legs*: Appearing speckled with mixture of whitish-buff and gray-buff. Tibiae without spiniform setae. Tarsi with three ventral rows of spiniform setae. *Wings*: Dorsal forewing pale whitish buff brown with dusting of pale-brown scales; subbasal, antemedial and postmedial lines very faint, indicated by paler lines bordered on each side by scattered pale-brown scales; subterminal line more distinct because of darker shading in terminal area and outer part of subterminal area adjacent to it; reniform and orbicular spots slightly darker than ground color, outlined in white; terminal area darker than remainder of forewing because of more numerous gray-brown scales; fringe similar in color to subterminal area with base of fringe whitish-buff. Dorsal hindwing white, translucent, with trace of darker scaling on veins and wing margin; fringe white with scattered pale fuscous scales. **Male genitalia** – Similar to those of *Protorthodes texicana* with following exceptions: lower process of clasper apically flattened and enlarged, spatulate (apically tapered and tear-drop shaped in *Protorthodes texicana*); spine-covered dorsal process of sacculus acutely tapered to point (posterior apex blunt and rounded in *Protorthodes texicana*); vesica with two coils (1½ coils in *Protorthodes texicana*).

##### Distribution and biology.

The female and immature stages are unknown. The species is known only from the type locality where the holotype was collected in late April.

#### Protorthodes perforata

Taxon classificationAnimaliaLepidopteraNoctuidae

(Grote, 1883)

Figs 31 , 32 , 65 , 82 ; Map 9

Taeniocampa perforata Grote, 1883: 73.
Eriopyga constans Dyar, 1918: 344, **syn. n.**

##### Type material.

*Taeniocampa perforata*: syntypes, USNM, examined. Type locality: Arizona. *Eriopyga constans*: holotype ♂, USNM, examined. Type locality: Mexico.

##### Diagnosis.

*Protorthodes perforata* can be recognized by the pale whitish gray to buffy gray color of the forewings with the reniform and orbicular spots darker gray than the ground color and each is outlined by a contrastingly pale line. Forewing length varies from 12 to 14 mm. The hindwing is dirty white with fuscous shading on the wing margins and veins in both sexes. This species is most likely to be confused with *Protorthodes rufula* in southern California where their ranges overlap. The forewing in *Protorthodes rufula* is darker and more mottled than that of *Protorthodes perforata*, and *Protorthodes rufula* usually has shades of red in the ground color. Males of *Protorthodes perforata* can readily be distinguished from those of *Protorthodes rufula* by brushing the scales away from the apices of the valves; in *Protorthodes perforata* the apices of the digitus on each valve are similar, barely extend beyond the ventral margins of the valve, and the apices curl to project anteroventrally. In *Protorthodes rufula*, by contrast, the left digitus projects posteroventrally, whereas the right digitus is much longer and projects posteriorly and extends beyond the apex of the right valve (Fig. 66). On dissection, the valves of *Protorthodes perforata* are very wide postmedially with both the dorsal and ventral margins rounded and convex; the ampulla of the clasper is almost straight, bending posteriorly only near the apex; the heavily-sclerotized dorsal lobe of the sacculus is much larger and more convex than in *Protorthodes rufula*; and the large cornutus in the vesica is near the middle, whereas in *Protorthodes rufula* it is much closer to the base of the vesica; also the cornutus projects at a right angle to the axis of the vesica and parallel to the aedeagus. In the female genitalia of *Protorthodes perforata* the sclerotized mass of the ostium bursae covers almost the entire ductus bursae and is rounded anteriorly.

##### Distribution and biology.

*Protorthodes perforata* occurs across the southern United States from western Texas to southern California and southward to central Mexico. According to material examined, the range of *Protorthodes perforata* lies to the east of that of *Protorthodes rufula* with the westernmost records of *Protorthodes perforata* being from the desert areas in interior southern California. Adults were collected from early April to early July and again from mid-August to late October. The immature stages are unknown.

#### Protorthodes rufula

Taxon classificationAnimaliaLepidopteraNoctuidae

(Grote, 1874)

Figs 33 –38 , 66 , 83 ; Map 10

Dianthoecia rufula Grote, 1874a: 64.

##### Type material.

*Dianthoecia rufula*: holotype ♀, BMNH, examined. Type locality: California, Oakland.

##### Diagnosis.

*Protorthodes rufula* is much more varied in color than *Protorthodes perforata* with the forewing varying from pale whitish buff, through various shades of red and orange, to brown. Unlike in *Protorthodes perforata*, almost all specimens show some areas or patches of rufous shading and the ground color is much more mottled in *Protorthodes rufula*. Forewing length ranges from 13 to 16 mm, averaging larger than in *Protorthodes perforata*. In the male genitalia, the right digitus projects posteriorly beyond the end of the valve, whereas on the left valve it is shorter and projects posteroventrally below the valve; the ampulla of the clasper is evenly curved in a rounded arc on both valves; the dorsal lobe of the sacculus is elongated and narrow with the dorsal margin slightly concave and extending into a rounded densely setose lobe anteriorly. The vesica has a long cornutus about 1/3 from the base that projects along the axis of the vesica toward the end of the aedeagus. In the female genitalia the ostium bursae is covered by a heavily-sclerotized ventral plate that is lobed and convex posteriorly and concave where it meets the ductus bursae anteriorly; the ductus bursae is elongated, about 2 × as long as the ostial plate, and is sclerotized with elongated ridges and folds.

**Figures 37–54.**
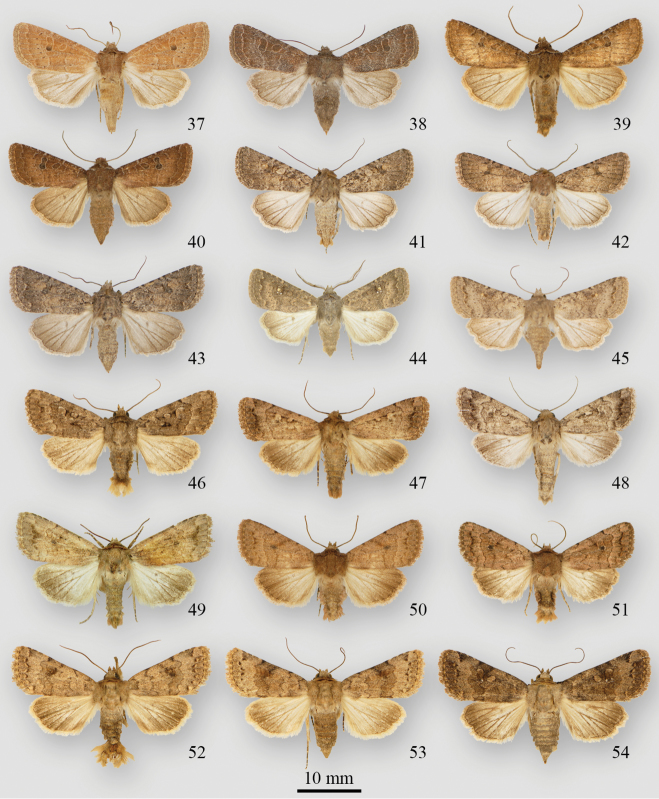
*Protorthodes* and *Nudorthordes* adults **37** *Protorthodes rufula* ♀, USA, California, Sonoma Co., Petaluma **38** *Protorthodes rufula* ♀, USA, California, San Diego Co., Mira Mesa at Penasquitos Canyon **39** *Protorthodes ustulata* ♂ paratype, USA, Colorado, Larimer Co., Viestenz-Smith Mtn Park W of Loveland, 5700’ **40** *Protorthodes ustulata* ♀, USA, New Mexico, Grant Co., Signal Peak, 8020’, 32°55.62’N, 108°09.91 W **41** *Protorthodes alfkenii* ♀, USA, California, San Bernardino Co., San Bernardino Mts, Cactus Flats, 6100’ **42** *Protorthodes alfkenii* ♂, USA, Arizona, Cochise Co., Huachuca Mts, Carr Canyon, 5600’ **43** *Protorthodes alfkenii* ♀, USA, California, Ventura Co., Cuyama Valley, Apache Canyon **44** *Protorthodes antennata* ♂, USA, Arizona, Pima Co., Madera Canyon, 4600’ **45** *Protorthodes antennata* ♀, USA, Arizona, Pima Co., Baboquivari Mts **46** *Nudorthodes texana* ♂, USA, Texas, San Patricio Co., Sinton **47** *Nudorthodes texana* ♂, USA, Utah, Grand Co., Sego Canyon, 5900’, 39°03.00'N, 109°43.42'W **48** *Nudorthodes texana* ♂, USA, Nevada, Humboldt Co., Winnemucca Mts, 5600’ **49** *Nudorthodes texana* ♂, USA, California, Yuma Co., Yuma **50** *Nudorthodes variabilis* ♂, USA, California, Los Angeles Co., La Tuna Canyon **51** *Nudorthodes variabilis* ♂, USA, California, San Diego Co., Mira Mesa **52** *Nudorthodes molino* holotype ♂, USA, Arizona, Pima Co., Santa Catalina Mts, mi 5.5 Mt. Lemon Hwy, 4400’ **53** *Nudorthodes molino* paratype ♀, USA, Arizona, Pima Co., Baboquivari Mts, Brown Canyon, 4100’ **54** *Nudorthodes molino* paratype ♀, USA, Arizona, Santa Catalina Mts, mi 5.7 Mt. Lemon Hwy, 4400’.

**Figures 55–60.**
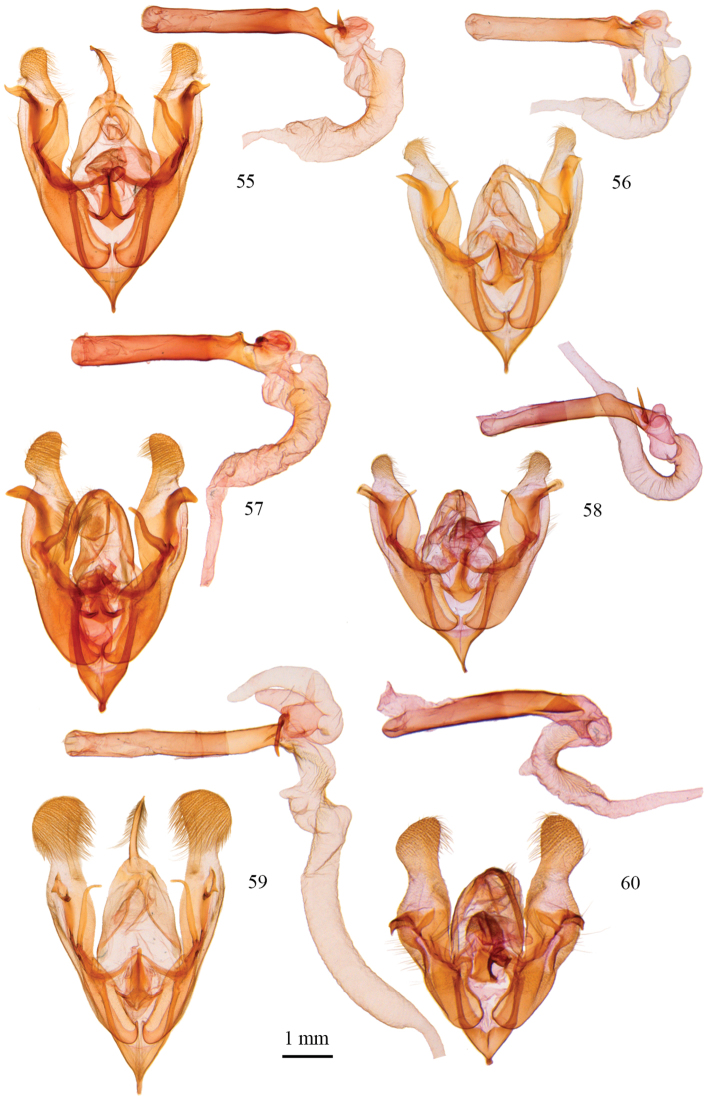
*Protorthodes* male genitalia. **55** *Protorthodes curtica*; CNC slide 15555 **56** *Protorthodes eureka*; CNC slide 10889 **57** *Protorthodes incincta*; CNC slide 15327 **58** *Protorthodes argentoppida*; CNC slide 15237 **59** *Protorthodes mulina*; CNC slide 15576 **60** *Protorthodes oviduca*; CNC slide 15238.

**Figures 61–65.**
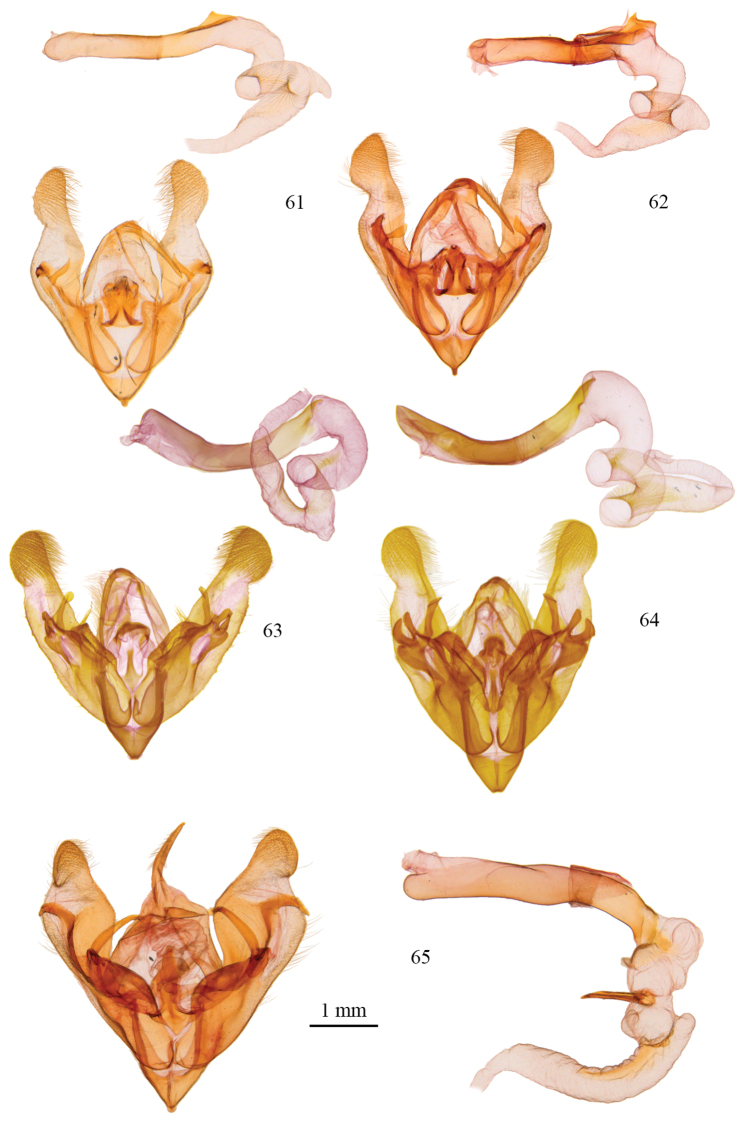
*Protorthodes* male genitalia. **61** *Protorthodes orobia*; CNC slide 15580 **62** *Protorthodes melanopis*; CNC slide 11603 **63** *Protorthodes texicana*; CNC slide 15658 **64** *Protorthodes mexicana*; CNC slide 11593 **65** *Protorthodes perforata*; CNC slide 11478.

**Figures 66–69.**
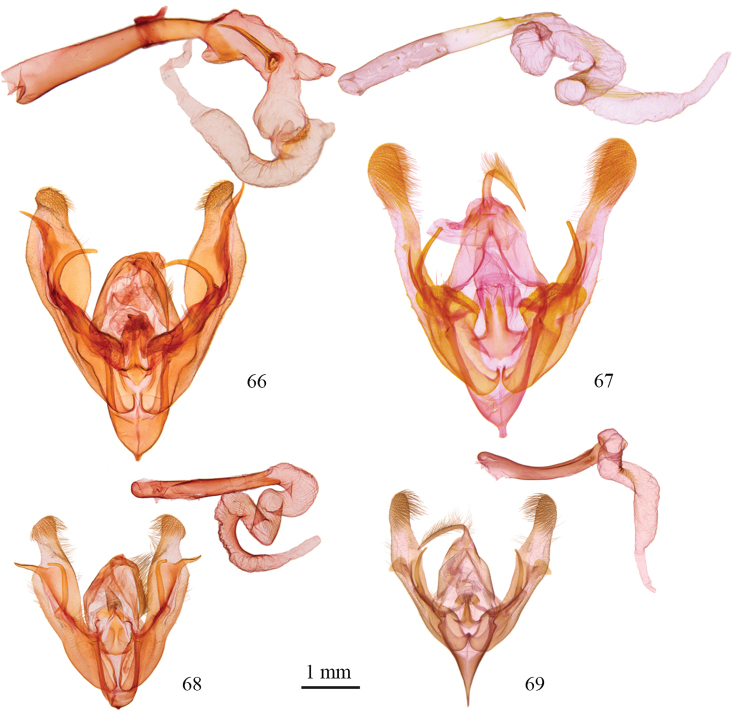
*Protorthodes* male genitalia. **66** *Protorthodes rufula*; CNC slide 11476 **67** *Protorthodes ustulata*; CNC slide 16599 **68** *Protorthodes alfkenii*; CNC slide 11788 **69** *Protorthodes antennata*; CNC slide 16608.

##### Distribution and biology.

*Protorthodes rufula* is a species of the far west with most records being from the Pacific Coast, and the coastal mountain ranges from northern Washington to southern California – to the west of the known range of *Protorthodes perforata* in southern California. Adults were collected from mid-April to mid-June in the North (from mid-February in southern California) and again from early August to late October. The larva was described by Crumb (1956) and Godfrey (1972).

#### Protorthodes ustulata

Taxon classificationAnimaliaLepidopteraNoctuidae

Lafontaine, Walsh & Ferris sp. n.

http://zoobank.org/49452489-03F9-4654-8799-C900EE7692C2

Figs 39 , 40 , 67 , 84 ; Map 11

##### Type material.

**Holotype** ♂. USA, Arizona, Cochise Co., [Chiricahua Mts], Pinery Canyon, 7000’, 31°55.99'N, 109°16.33'W, 22 April 2007, C.D. Ferris. CNC. **Paratypes:** 29 ♂, 29 ♀. Type series restricted to specimens from Arizona. Same data as for holotype (1 ♂, 2 ♀); Arizona, Cochise Co., [Chiricahua Mts], Pinery Canyon, 6000’, 31°56.89'N, 109°18.40'W, 13 April 2005, C.D. Ferris (3 ♀); Arizona, Cochise Co., Chiricahua Mts, Southwestern Research Station, 8–25 April 1962 (14 ♂, 7 ♀), 18 Sept. 1962 (1 ♀), Carl Kirkwood; Arizona, Cochise Co., [Huachuca Mts], Carr Canyon, 5600’, 31°26.38'N, 110°15.87'W, 11 Oct. 2006 (1 ♀), Barcode of Life sample ID # CNCNoctuoidea12245; Arizona, Cochise Co., [Huachuca Mts], Carr Canyon, 5600’, 31°26.38'N, 110°17.35'W, 2 Oct. 2004, 10 April 2005, C.D. Ferris (2 ♂); Arizona, Cochise Co., Huachuca Mts, Garden Canyon, 4 Aug. 1966 (1 ♂), 17 Sept. 1967 (1 ♂), R.F. Sternitzky; Arizona, [Cochise Co.], Huachuca Mts, Ramsey Canyon, 24 April 1965 (1 ♀), 17 Sept. 1967 (1 ♂), R.F. Sternitzky; Arizona, [Cochise Co.], Huachuca Mts, Ramsey Canyon, 6000’, 15 mi SW Sierra Vista, 5100’, 13 May 1967 (2 ♀), R.F. Sternitzky; Arizona, [Cochise Co.], Sierra Vista, 20 Aug. 1966 (1 ♂), 20 Sept. 1967 (1 ♂), R.F. Sternitzky; Arizona, Cochise Co., Huachuca Mts, 5354 Ash Canyon Rd, 0.5 mi W Hwy 92, 15 Sept. 1999, Noel McFarland (1 ♀); Arizona, Pima Co., Madera Canyon, 4600’, 23 Sept. 1986, D.F. Hardwick (1 ♀); Arizona, Pima Co., Santa Catalina Mts, Bear Canyon, 4800’, [NE of] Tucson, 7 Oct. 2006, Ian A. Watkinson (1 ♀); Arizona, Yavapai Co., 4 mi N Prescott, 31 Aug. 1970 (1 ♂), 8 Sept. 1970 (1 ♂), 25 Aug. 1972 (2 ♀), 9 Sept. 1972 (1 ♀), Lloyd M. Martin; Arizona, [Yavapai Co.], Jerome, 4 mi SSW, 7000’, 20 Sept. 1970, D.F. Hardwick (2 ♀); Arizona, Flagstaff, [Coconino Co.], 6850’, 31 Aug. 2007, Ian A. Watkinson (1 ♀); Arizona, Apache Co., White Mts, Diamond Rock, 5–6 Sept. 1947, G.H. & J.L. Sperry (4 ♂, 1 ♀); Arizona, Apache Co., 3 mi W Eagar, 7100’, 11 Aug. 1962, E. & I. Munroe (1 ♀); Arizona, Apache Co., Alpine, 8117’, 31 Aug. 2003, Ian A. Watkinson (1 ♂, 1 ♀);. Paratypes deposited in CDF, CNC, IAW, JBW, USNM.

##### Etymology.

The name *ustulata* is Latin and refers to the burnt-orange color of with body and forewings of the species.

##### Diagnosis.

*Protorthodes ustulata* looks like a dark burnt-orange form of *Protorthodes perforata* and was usually identified as either *Protorthodes perforata* or as *Protorthodes oviduca* in collections because of the orange in the ground color and the contrasting pale outlines around the reniform and orbicular spots. The species appears to be most closely related to *Protorthodes perforata* and *Protorthodes rufula*, not just because of the superficial similarity, but because of the heavily-sclerotized spiculate lobe on the dorsal part of the sacculus, a structure restricted to these three species. Beyond that, however, *Protorthodes ustulata* is unique within the genus in several features: in the male genitalia the valves are very long and slender and the apices easily broken in brushing or dissection; the digitus is vestigial, ending in small lobe free of surface of valve at ventral margin of valve; the spine-covered dorsal lobe on the sacculus is oblique to the longitudinal axis of the valve. In the female genitalia, the ostium bursae and posterior part of the ductus bursae form an elongated, narrow, heavily-sclerotized tube, and the appendix bursae has two coils, both features otherwise found only in *Protorthodes alfkenii*.

##### Description.

**Adult.** Male and female similar in size, color, and maculation. Forewing length: 12–15 mm. **Head** – Male antenna biserrate with projections (rami) tapered, anterior rami about as long as width of central shaft, posterior rami half as long. Female antenna filiform, minutely setose ventrally. Palpi and head clothed with spatulate apically-forked setae, dark orange or brown, some with white tips giving head a hoary look, white tips more frequent on margin of prothoracic collar (patagia). **Thorax** – Covered with similar scales to those of head; with slightly raised prothoracic and metathoracic tufts. *Legs*: Appearing speckled with mixture of dark-orange, brown scales, some white tipped. Tibiae without spiniform setae. Tarsi with three ventral rows of spiniform setae. *Wings*: Dorsal forewing a burnt-orange color, tending to be darker toward costal and outer edge of forewing; subbasal, antemedial, postmedial, and subterminal lines whitish gray, partially bordered by dark-brown scales; reniform spot kidney shaped, to markedly constricted mesially, so almost figure 8 shaped, upper part gray brown, lower part blackish gray, overall darker than forewing and with contrasting whitish-gray outline; orbicular spot obliquely oval, slightly darker than ground color, outlined in whitish gray; terminal line dark brown; fringe similar in color to forewing except base yellow with fine yellow streak at end of each wing vein. Dorsal hindwing pale fuscous basally with darker fuscous on discal spot, wing veins, and marginal area of wing; fringe buff white with fuscous medial line. **Male genitalia** – Uncus slender, evenly tapered from base to apex, sparely covered with long hair-like setae, apex tapered to point. Valve elongated and narrow, about 5 × as long as width at base of clasper, gradually narrowing to long “neck” before rounded cucullus densely covered with long, stiff setae projecting anterodorsally, with no apical corona; sacculus sclerotized, about 0.4 × length of valve, ending posteriorly in spine-covered oblique lobe, somewhat mushroom shaped, with sides extending posteroventrally almost to ventral margin of valve and anterodorsally over dorsal margin of valve; clasper about 1/3 length of valve, curved in slight arc to project posterodorsally over dorsal margin of valve; digitus vestigial, a slightly raised, lightly sclerotized rod extending to base of “neck” of cucullus and ending in slightly raised lobe projecting posteroventrally to ventral margin of valve; juxta a long posteriorly-tapered plate with middle cleft and lightly sclerotized almost to anterior end, posterior apices on each side of cleft pointed and heavily sclerotized. Aedeagus long and cylindrical, 12 × as long as medial width, mainly lightly sclerotized except for posterior half of ventral margin, which ends in slightly-sclerotized lobe. Vesica about 1.5 × as long as aedeagus; vesica with slight basal coil from which arises a curved, tapered diverticulum, and a medial coil with a minute diverticulum; apex of vesica swollen; vesica without spines or cornuti. **Female genitalia** – Corpus bursae membranous, rounded, without signa. Appendix bursae with two short coils, arising from posterior dorsal surface of corpus bursae. Ductus bursae 0.6 × as long as corpus bursae, anterior 1/3 membranous, posterior 2/3 covered with smooth heavily-sclerotized plate, slightly wider anteriorly than posteriorly; ostium not differentiated from ductus bursae; abdominal segment eight slightly longer than wide, more lightly sclerotized posteriorly; anterior apophyses slightly longer than abdominal segment eight; posterior apophyses folding near middle, about 2 × as long as anterior apophyses. Anal papillae long and tapered, 0.75 × as long as abdominal segment eight; anal papillae lightly sclerotized, long setae sparsely scattered over surface, short setae abundant near apex of papillae.

##### Distribution and biology.

*Protorthodes ustulata* occurs from southeastern Wyoming southward to the Guadalupe Mountains in western Texas and westward to central and southeastern Arizona and northern Mexico. Adults were collected from early April to mid-May and again from early August to early October. The immature stages are unknown.

#### Protorthodes alfkenii

Taxon classificationAnimaliaLepidopteraNoctuidae

(Grote, 1895)

Figs 41–43 , 68 , 85 ; Map 12

Perigea alfkenii Grote, 1895: 79.
Perigea latens Smith, 1908a: 92.
Taeniocampa occluna Smith, 1909: 64.

##### Type material.

*Perigea alfkenii*: syntypes, 1 ♂ male, 2 ♀ females, USNM, examined. Type locality: [southwestern USA]. *Perigea latens*: lectotype ♂, USNM, examined. Type locality: California, San Diego. *Taeniocampa occluna*: holotype ♂, USNM, examined. Type locality: New Mexico, Mesilla Park.

##### Note.

The name *Perigea perplexa* was listed without description by Smith (1893) and credited to Grote by virtue of Grote distributing the name through his check lists, which lacked any description. Grote described *Perigea alfkenii* in 1895 and lists *Perigea perplexa* as his check list manuscript name for it. Hampson (1909) lists *Perigea perplexa* as an unavailable senior synonym of *Perigea alfkenii* and credits the name to Smith. Franclemont and Todd (1983) simply list *Protorthodes* [*Perigea*] *perplexa* (Grote, 1895) as a synonym of *Protorthodes alfkenii*. The name, neither validated, nor made available, should be deleted from the synonymy.

##### Diagnosis.

*Protorthodes alfkenii* is an extremely variable species in terms of size, ground color, and pattern, yet with practice it usually is easily identified by a combination of features. The orbicular spot usually is rounded, surrounded by a thin black line, and the spot itself usually is paler than the ground color, often contrasting so; the reniform spot is oblique, unlike other species in the genus, with the lower part of the spot projecting toward the anal angle of the wing; the light and dark marks on the forewing, and the tendency for longitudinal streaks on the wing, give the forewing a busy appearance instead of the softer, more even ground pattern of most other species. The hindwing in males, and many females, is white, often with a slight pearly sheen, with fuscous shading confined to the veins and outer part of the wing. Some females have more extensive fuscous shading on the hindwings, but usually a pearly sheen is still evident. Forewing length varies from 11 to 14 mm. The male genitalia differ from those of other species of *Protorthodes* in that the digitus is long and pointed, projecting below the ventral margin of the valve at a right angle, the ampulla of the clasper is almost straight, projecting dorsoposteriorly toward the upper part of the cucullus, then bending abruptly through 90°near its apex to project ventrally. The vesica has 2½ medial coils and projects anterolaterally to the right from the end of the aedeagus. In the female genitalia the appendix bursae has two full coils and is at the posterior end of the corpus bursae on the left side. The ductus bursae is long and cylindrical, about 7 × as long as wide with the posterior 4/5^th^ lightly sclerotized.

##### Distribution and biology.

*Protorthodes alfkenii* is the most abundantly collected species in the American Southwest, including southern California. It occurs from central Oregon, southern Idaho, central Wyoming, and northwestern Texas southward to southern Mexico. It occurs mainly in open arid woodlands and although its range surrounds the Great Basin, it is largely absent from the Basin. Adults were collected in the South from April until late June and again from early September to early November. In the Pacific Northwest they fly mid-July to late September. The larva was described by Crumb (1956) and Godfrey (1972).

#### Protorthodes antennata

Taxon classificationAnimaliaLepidopteraNoctuidae

(Barnes & McDunnough, 1912)

**Maps 13–16.**
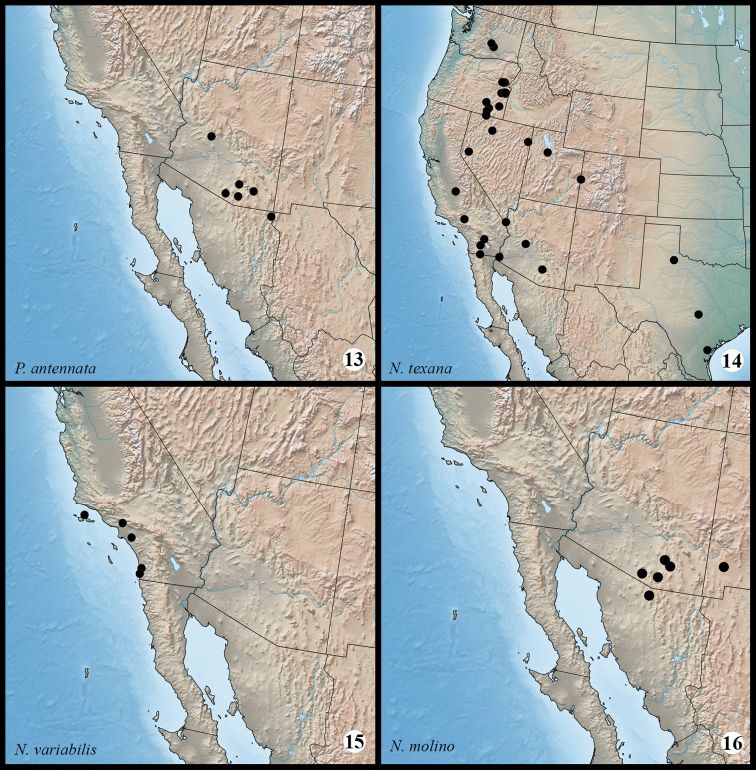
Distribution of examined material for *Protorthodes* and *Nudorthodes* species. **13** *Protorthodes antennata* **14** *Nudorthodes texana* **15** *Nudorthodes variabilis* **16** *Nudorthodes molino*.

Figs 44 , 45 , 69 , 86 . Map 13

Eriopyga antennata Barnes & McDunnough, 1912a: 21.

##### Type material.

Syntypes 4 ♂, 3 ♀, USNM, examined. Type locality: Arizona, Redington.

##### Diagnosis.

Males of *Protorthodes antennata* can easily be identified by the very long pectinations of the antennae, which gives them a feathery appearance. The other diagnostic feature, which applies to females as well, is the form of the reniform spot. In *Protorthodes antennata* the reniform spot is not outlined like in other species of *Protorthodes*; there is a series of tiny white dots that partially define the reniform spot, and a series of tiny yellow dots that form a partial outer border of the spot. Forewing length varies from 10 to 14 mm with females tending to be larger than males. The male genitalia are characterized by the short, broad, apically-truncated digitus, and the very narrow, posteriorly tapered juxta. The apex of the aedeagus has a double field of flattened scale-like sclerites that create a lizard-skin appearance. In the female genitalia, the ductus bursae is inflated mesially and covered with minute sclerotized spicules; the ductus is mainly membranous except for a narrow sclerotized “collar” at the posterior end.

##### Distribution and biology.

*Protorthodes antennata* is rarely collected but can occasionally be locally common. It has a small distribution extending from central Arizona to northernmost Mexico. Adults seem to have an abbreviated flight period between mid-May and mid-June, and in October. The immature stages are unknown.

#### Nudorthodes

Taxon classificationAnimaliaLepidopteraNoctuidae

Lafontaine, Walsh & Ferris gen. n.

http://zoobank.org/81DE3680-4B3E-456B-B7E9-D2FA578487CA

##### Type species.

*Perigea texana* Smith, 1900: 476.

##### Etymology.

The generic name *Nudorthodes* is a reference to this species being removed from the other members of the *Orthodes* group of genera by the lack of hairs on the surface of the eye. From the Latin *Nudus* [bare] + *Orthodes*.

##### Diagnosis.

The genus *Nudorthodes* differs from other genera in the Hadenini: Eriopygina in lacking visible hair-like setae on the surface of the eye, and by the very long vesica in males and appendix bursae in females. Males can be distinguished from those of most other eriopygine genera by the filiform antennae. The genus includes three species: *Nudorthodes texana* (Smith, 1900), *Nudorthodes variabilis* (Barnes & McDunnough, 1912), and *Nudorthodes molino* Lafontaine, Walsh, & Ferris, sp. n.

##### Description.

**Adult: Head** – Frons rounded; labial with apical segment about 1/4 as long as second segment; male antenna filiform, or very slightly constricted between segments (*Nudorthodes variabilis*), setose ventrally; female antenna filiform, setose ventrally; eye rounded, without surface hairs; ocellus present. **Thorax** – Thorax clothed with narrow, spatulate, apically serrated scales that form a slightly raised tuft on the prothorax, and a partially divided tuft on the metathorax. *Legs*: middle and hind tibiae without spiniform setae and with three ventral rows of spiniform setae on tarsi. *Wings*: forewing venation typically quadrifine, cubital vein appearing four branched; hindwing with typical trifine venation (i.e., M2 reduced, about 2/3 down cell and parallel to M3. **Abdomen** – basal abdominal brushes and pockets absent; eighth abdominal sternite of male with a slightly eversible coremata with a transverse tuft of long setae. **Male genitalia** – *Valva*: symmetrical, basal ¾ of valve mainly parallel-sided except for dorsal lobe of sacculus (basal half of valve, mainly sacculus, massive compared to narrow apical half in *Nudorthodes molino*); valve slightly constricted at ¾ from base to define a tapered densely setose cucullus with much stouter setae on apical and ventro-apical part of cucullus forming an irregular corona; digitus projecting posteriorly along middle of valve, bending ventro-posteriorly near base of cucullus and flattened into elongated plate extending beyond ventral margin of valve (apical part of digitus slightly s-curved and flattened into a foot-shaped structure in *Nudorthodes molino*); clasper arising as a sclerotized rod at apex of sacculus projecting posteriorly with ampulla bending abruptly through 45°angle to project posterodorsally beyond costal margin of valve; sacculus heavily sclerotized, with a large dorsal process (lobe somewhat quadrangular in two species, and produced posteriorly in *Nudorthodes molino*), without membranous flap; *Uncus*: decurved, cylindrical, tapered apically to a down-curved sharply pointed apex. *Aedeagus*: long and slender, about 7 × as long as wide, dorsal surface mostly membranous, lateral margins extended on to base of vesica; everted vesica tubular, about 4–5 × as long as aedeagus, basal area angled and with several diverticula, main part gently curved through 5 or 6 coils, vesica expanded and covered with short sclerotized spine-tipped granules toward apex. **Female genitalia** – Corpus bursae rounded, membranous, without signa and with inner surface covered with minute spicules. Appendix bursae arising from left posterior part of corpus bursae and extending anteriorly through several open coils; appendix bursae about 2 × as long as corpus bursae. Ductus bursae about at long as corpus bursae, mainly membranous, with a sclerotized collar-like ring at posterior end forming a slightly wider ostium bursae. Abdominal segment eight sclerotized, covered posteriorly with numerous long, heavily-sclerotized setae, many half as long as segment. Anterior apophyses rod-like, 1.2–1.4 × as long as abdominal segment eight. Posterior apophyses 1.6–1.9 × longer than anterior apophyses. Anal papillae lightly sclerotized, bullet shaped, gradually tapered to rounded apex; surface covered with long hair-like setae, especially toward apex.

##### Larva and habits.

The only species of *Nudorthodes* known as a larva is *Nudorthodes texana*. Like *Protorthodes* the species are generally associated with xeric habitats. The larvae were described by Crumb (1956) and Godfrey (1972), and were separated from *Protorthodes* by both authors and described as being more similar to larvae of *Homothodes* McDunnough. The larva of *Nudorthodes texana* differs from those of *Protorthodes* species in that the larval skin is smooth, not granulose, the setae arise from sclerotized rings, not pinacula, the posterior part of the prothoracic shield is not contrastingly pale, sclerotized plates between the bases of the abdominal prolegs are lacking, and the apical seta of the labial palpus (Lp-2) is similar in length to the basal segment of the palpus (Lps-1).

##### Distribution.

USA (Arizona, California, Nevada, Oregon, Texas, Utah, Washington).

#### Nudorthodes texana

Taxon classificationAnimaliaLepidopteraNoctuidae

(Smith, 1900) comb. n.

**Figures 70–72.**
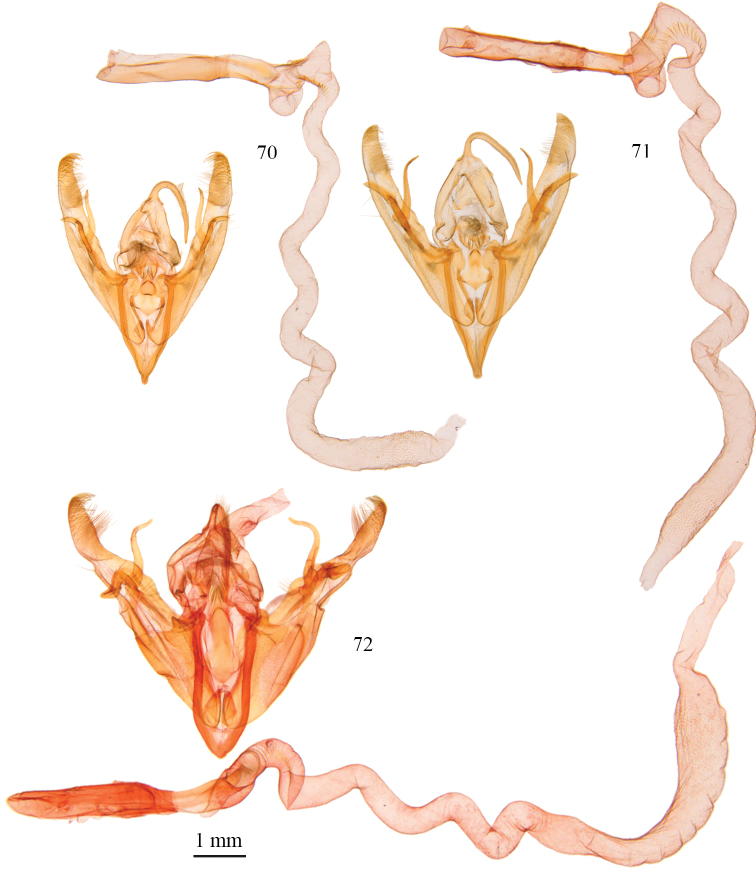
*Nudorthordes* male genitalia. **70** *Nudorthodes texana*; CNC slide 15581 **71** *Nudorthodes variabilis*; CNC slide 15583 **72** *Nudorthodes molino*; CNC slide 14211.

Figs 46–49 , 70 , 87 ; Map 14

Perigea texana Smith, 1900: 476.
Perigea consors Smith, 1900: 477.

##### Type material.

*Perigea texana*: lectotype ♂, USNM, designated by Todd (1982), examined. Type locality: USA, Texas, Round Mountain. *Perigea consors*: lectotype ♂, USNM, designated by Todd (1982), examined. Type locality: USA, Phoenix, Arizona.

##### Diagnosis.

*Nudorthodes texana* is the most widely distributed and common species in the genus. In most of its range adults can be recognized by the pale buffy-brown or gray-brown color of the forewings with darker shading around the reniform and orbicular spots and in the outer part of the subterminal area. The male and female genitalia of *Nudorthodes texana* form the basis for the description of the genus *Nudorthodes*; it and *Nudorthodes variabilis* are structurally very similar, differing mainly in the shape of the costal process of the sacculus and the relative lengths of the ampulla of the clasper in the male genitalia. The barcode of *Nudorthodes texana* is most similar to that of *Nudorthodes variabilis*, the two differing by 3.67–3.98 %.

**Figures 73–81.**
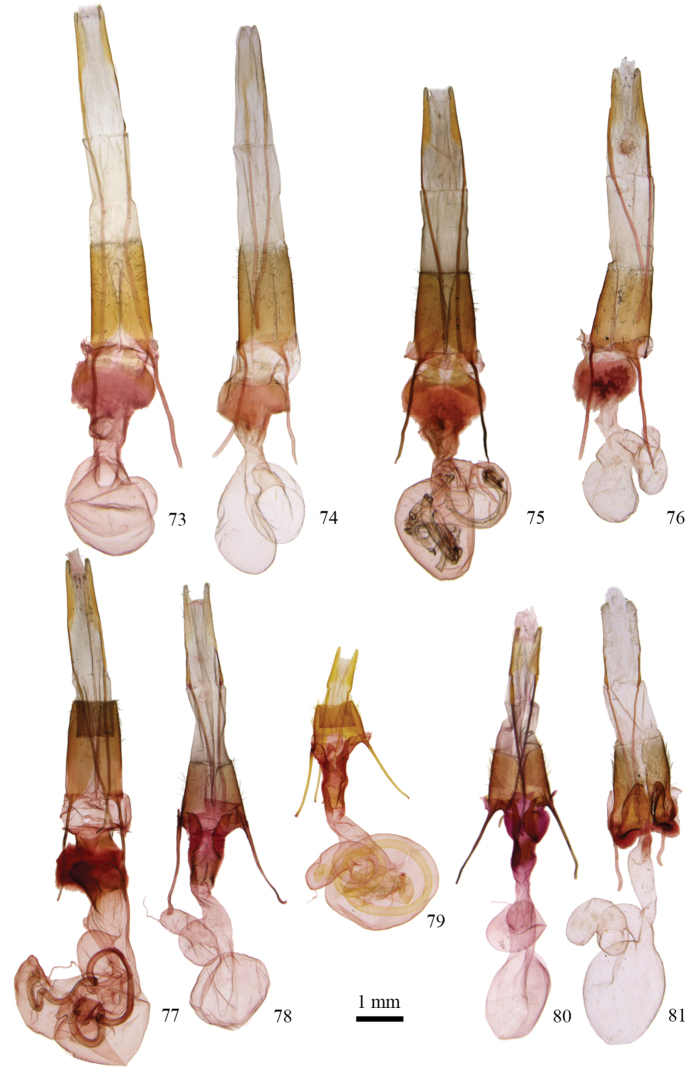
*Protorthodes* female genitalia. **73** *Protorthodes curtica*; CNC slide 16552 **74** *Protorthodes eureka*; CNC slide 15255 **75** *Protorthodes incincta*; CNC slide 15562 **76** *Protorthodes argentoppida*; CNC slide 15253 **77** *Protorthodes mulina*; CNC slide 15577 **78** *Protorthodes oviduca*; CNC slide 15245 **79** *Protorthodes orobia*; CNC slide 11604 **80** *Protorthodes melanopis*; CNC slide 16004 **81** *Protorthodes texicana*; CNC slide 15254.

**Figures 82–89.**
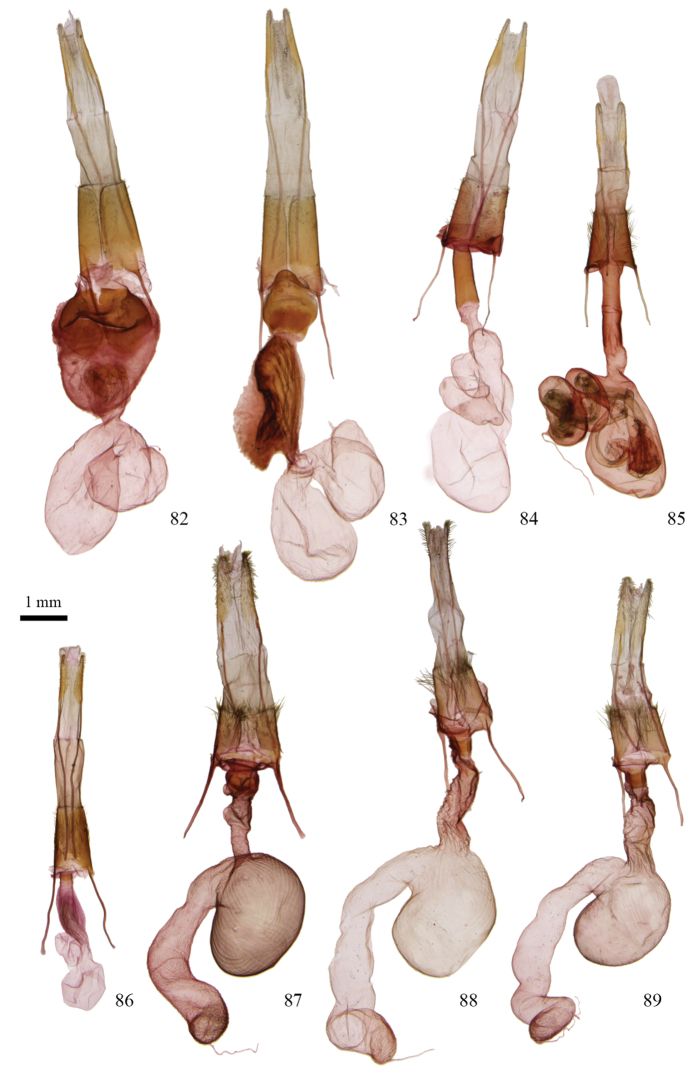
*Protorthodes* and *Nudorthordes* female genitalia. **82** *Protorthodes perforata*; CNC slide 15244 **83** *Protorthodes rufula*; CNC slide 15241 **84** *Protorthodes ustulata*; CNC slide 15246 **85** *Protorthodes alfkenii*; CNC slide 10927 **86** *Protorthodes antennata*; CNC slide 16605 **87** *Nudorthodes texana*; CNC slide 15239 **88** *Nudorthodes variabilis*; CNC slide 15251 **89** *Nudorthodes molino*; CNC slide 15240.

##### Distribution and biology.

*Nudorthodes texana* occurs from the intermontane region of Washington, Oregon, Nevada, and Utah southward to southern California and Arizona and southeastward to the Gulf Coast of Texas. Adults were collected rarely in the spring, mostly in March; the second generation occurs from mid-August to mid-November with the flight earlier in the North than in the extreme South. The larva was described by Crumb (1956) and Godfrey (1972).

##### Notes.

A peculiar form of *Nudorthodes texana* (Fig. 49) occurs in southwestern Arizona in the vicinity of Yuma. The forewing is a mottled pale orange brown or reddish brown, without the dark shading between the reniform and orbicular spots characteristic of *Nudorthodes texana*. The barcodes of this population differ from those of *Nudorthodes variabilis* by 3.36–3.67 % and from those of *Nudorthodes texana* by 4.43–5.05 %. However, many specimens from the Yuma area are intermediate in appearance between the mottled-orange form and the normal form of *Nudorthodes texana* and these barcode with either haplotype clade, suggesting adults of the two haplotype groups are interbreeding, or did so in the past. For this reason we do not describe the Yuma form as a distinct species, but suggest more research on the two haplotype groups in this area is needed. Only five specimens of the deviant haplotype are known.

#### Nudorthodes variabilis

Taxon classificationAnimaliaLepidopteraNoctuidae

(Barnes & McDunnough, 1912) comb. n.

Figs 50 , 51 , 71 , 88 ; Map 15

Namangana variabilis Barnes & McDunnough, 1912b: 21.

##### Type material.

*Namangana variabilis*: syntypes, USNM, examined. Type locality: USA, California, San Diego.

##### Diagnosis.

*Nudorthodes variabilis* is a rarely collected species confined to coastal southern California. Adults are most likely to be confused with those of *Nudorthodes texana*, but average larger (forewing length: 13 to 14 mm). In *Nudorthodes variabilis*, the medial line usually is prominent, extending obliquely from the costa to the reniform spot and as a straight line from there to the hind margin of the forewing, and the lower third of the reniform spot is filled with a well-defined dark blue-gray patch. In *Nudorthodes texana* the medial line is absent or barely traceable, and the lower third of the reniform spot has diffuse dark shading. In southern California where the ranges of the two species overlap, the forewing ground color in *Nudorthodes variabilis* is an even gray brown, sometimes with a slight reddish tint; whereas in *Nudorthodes texana* the ground color is pale buff and powdery in appearance. In the male genitalia the dorsal lobe on the sacculus of *Nudorthodes variabilis* is produced posteriorly towards the top, giving it a slight mushroom shape, whereas the posterior margin of the lobe in *Nudorthodes texana* is straight and perpendicular to the longitudinal axis of the sacculus. The female genitalia of the two species appear to be indistinguishable.

##### Distribution and biology.

*Nudorthodes variabilis* occurs along the coast of southern California from Santa Barbara County to San Diego County. Adults were been collected from late August until mid-September. The immature stages are unknown.

#### Nudorthodes molino

Taxon classificationAnimaliaLepidopteraNoctuidae

Lafontaine, Walsh & Ferris sp. n.

http://zoobank.org/B5727D96-128D-44B4-A95A-93D7704CBFE9

Figs 52–54 , 72 , 89 ; Map 16

##### Type material.

**Holotype** ♂. USA, Arizona, Pima Co., Santa Catalina Mts., mi 5.5 Mt Lemmon Hwy., 4400’, riparian blue oak woodland, uv light trap, 27 October 2007, B. Walsh. CNC. **Paratypes:** 59 ♂, 34 ♀. USA, Arizona, Pima Co., Baboquivari Mts., Brown Canyon Nature Center,, 4200’, oak woodland/ocotillo forest, mv/uv light trap, 11 June 2004, B. Walsh (1 ♂, 2 ♀, 1 ♀ Barcode of Life sample ID # CNCNoctuoidea13977); USA, Arizona, Pima Co., Baboquivari Mts., Brown Canyon Nature Center, 4200’, 11 June, 2004, CD Ferris (1 ♂); USA, Arizona, Pima Co., Baboquivari Mts., Brown Canyon Nature Center, 4100’, oak/ocotillo forest, mv/uv light trap, 22 & 28 June 2005 & 10 Sept. 2004, B. Walsh (2 ♂, 3 ♀); USA, Arizona, Pima Co., Baboquivari Mts., Brown Canyon Nature Center, 4300–4400’, 28 June, 2005 (1 ♂), 18 June, 2008 (36 ♂, 1 ♀), CD Ferris; USA, Arizona, Pima Co., Santa Catalina Mts., mi 5.7 Mt Lemmon Hwy., 4400’, riparian/oak woodland, uv light trap, 18 Sept. 2003, 4 October 2003, 13 June 2005, B. Walsh (3 ♂, 2 ♀, 1 ♀ Barcode of Life sample ID # CNCNoctuoidea12245); USA, Arizona, Pima Co., Santa Catalina Mts., Molino Basin, mi 5.5 Mt Lemmon Hwy., 4400’, riparian blue oak woodland, uv light trap, 3 June 2004, B. Walsh (1 ♀); USA, Arizona Pima Co., Santa Catalina Mts., Molino Basin, mi 5.5 Mt Lemmon Hwy., 4400’, 26 September, 2003 (1 ♀); 24 May, 2004 (2 ♂); 3 June, 2004 (1 ♂), JB Walsh; USA, Arizona, Pima Co., Santa Catalina Mts., mi 5.5 Mt Lemmon Hwy., 4400’, riparian blue oak woodland, uv light trap, 29 May 2008, 27 October 2004, 8 November 2004 (Barcode of Life sample ID # CNCNoctuoidea12246), B. Walsh (1 ♂, 2 ♀); USA, Arizona, Pima Co., Rincon Mts., Happy Valley, Mescal Road 9 miles N Jct. Interstate 10, oak/riparian, 9 June 2005, B. Walsh (1 ♀); USA, Arizona, Pima Co., Santa Rita Mts., Madera Canyon, 4400’, 7 Sept. 1960 (1 ♂) and 4800’, 31 August 1959 (1 ♀), J. G. Franclemont; USA, Arizona, Santa Cruz Co., Peña Blanca Cyn., 3940’, 31°23.87'N, 111°05.61'W, 8 Sept. 2010, C.D. Ferris (5 ♂, 9 ♀); USA, Arizona, Santa Cruz Co., Walker Canyon E. of Peña Blanca Lake, 3885’, 12 June 2009, C.D. Ferris (1 ♀); Arizona, Santa Cruz Co., Peña Blanca Cyn., 4000’, 3 September, 2010, JB Walsh (1 ♀); USA, Arizona, Santa Cruz Co., Patagonia, 4040’, 13 June 2009, C.D. Ferris (1 ♀); USA, Arizona, Santa Cruz Co., Harshaw Rd., 4310’, 14 June 2008 (1 ♂), 12 June 2013 (1 ♂), CD Ferris; USA, Arizona, Cochise Co., Dragoon Mts., 4950’, 4 June, 2009, C.D. Ferris (1 ♂); USA, Arizona. Cochise Co., Huachuca Mts., Ash Canyon, 5170’, 16 June, 2008 (1 ♂), 20 June, 2008 (1 ♀), 7 June, 2009 (2 ♀), 6 June, 2010 (1 ♀), 9 June, 2010 (1 ♀), CD Ferris; USA, Arizona, Cochise Co., Mule Mts., Banning Creek, 5700’, 12 June, 2010 (1 ♀), CD Ferris; USA, New Mexico, Grant Co., Patterson Cyn., 5270’, 26 May, 2012, CD Ferris (1 ♂). Paratypes deposited in CDF, CNC, CUIC, JBW, USNM.

##### Etymology.

This species is named after the Molino Basin on Mt Lemmon where most of the type series was collected. The name is a noun in apposition.

##### Diagnosis.

*Nudorthodes molino* can be recognized by the diffuse dark-brown band across the outer third of the forewing between the medial line and the subterminal line, and the dark-brown shading of the medial line that fills most of the reniform spot. The male genitalia are characterized by the large triangular sacculus, the elongated oval cucullus, and the S-curved, foot-shaped digitus. The female genitalia are similar to those of *Nudorthodes texana* and *Nudorthodes variabilis*, but in *Nudorthodes molino* the ostium bursae is more rounded with the sides convex, whereas in *Nudorthodes texana* and *Nudorthodes variabilis* the ostium bursae is quadrangular with the sides parallel. The barcode of *Nudorthodes molino* is the most deviant of the species in the genus *Nudorthodes*, differing from the other species by as little as 5.05% (*Nudorthodes variabilis*) to as much as 5.81% (*Nudorthodes texana*).

##### Description.

**Adult.** Male and female similar in size, color, and maculation. Forewing length: 13–15 mm. **Head** – Male antenna filiform, setose ventrally. Female antenna filiform, ventral setae visible only under high magnification. Palpi and head clothed with spatulate apically-forked setae, pale buffy brown or gray brown. **Thorax** – Covered with similar scales to those of head; with slightly raised prothoracic and metathoracic tufts. *Legs*: Appearing speckled with mixture of buff and darker brown scales. Tibiae without spiniform setae. Tarsi with three ventral rows of spiniform setae. *Wings*: Dorsal forewing pale brown with dusting of darker-brown scales, especially in outer part of medial area, outer part of subterminal area, and usually in terminal area; subbasal, antemedial, postmedial lines dark brown, subterminal line a series of pale-buff dots with dark brown shading proximally that highlights line; reniform spot kidney shaped, infuscated with dark-brown shading from medial line; orbicular spot rounded, generally paler than ground color and outlined by thin dark-brown line; medial line variably expressed, usually forming a broad diffuse brown shading between it and postmedial line and filling most of reniform spot with dark shading; terminal line dark brown, sinuate; fringe with two irregular rows of spatulate scales, pale-brown at base, darker toward tips. Dorsal hindwing pale fuscous basally with darker fuscous toward wing margin without a well-defined discal spot; fringe mainly whitish buff with fuscous medial line. **Male genitalia** – Similar to generic description except as noted. Valves mainly symmetrical except dorsal process of sacculus, and ampulla of clasper larger on right valve than on left valve. Valve widest at 1/3 from base, abruptly tapered near middle, then gradually tapered to ¾ from base to “neck” of cucullus; cucullus densely covered with posterodorsally-directed hairlike setae on anterior half of cucullus with stout setae curving to project anterodorsally on posterior half of cucullus; digitus abruptly-angled ventrally near neck of cucullus with apical part curved, ventrally flattened and foot shaped; ampulla of clasper expanded and lobed at base, then gradually tapered to apex, abruptly curved through 90°angle at ¾ to project dorsally, right clasper about 15% larger than left one; dorsal process of sacculus elongated and lobed posteriorly, process larger on right valve than on left valve; juxta an elongated flattened plate extending into series of oblique sclerotized ridges in membrane directed toward midline. Aedeagus and vesica similar to those of *Nudorthodes texana* and other species but apical part of vesica more inflated. **Female genitalia** – Corpus bursae membranous, rounded, without signa; appendix bursae arising from posterior dorsal surface of corpus bursae, 1.5 × as long as corpus bursae and slightly coiled toward anterior end; ductus bursae about as long as corpus bursae, with expanded sclerotized ostium bursae on posterior ¼; abdominal segment eight as long as wide, lightly sclerotized; anterior apophyses 1.5 × as long as abdominal segment eight; posterior apophyses folding near middle, about 2 × as long as anterior apophyses. Anal papillae long and tapered, about as long as abdominal segment eight; anal papillae lightly sclerotized, long setae sparsely scattered over surface, short setae abundant near apex of papillae.

##### Distribution and biology.

*Nudorthodes molino* is known from southeastern Arizona and southwestern New Mexico. Adults were collected from late May until late June and from late August until early November. The immature stages are unknown.

## Supplementary Material

XML Treatment for Protorthodes

XML Treatment for Protorthodes curtica

XML Treatment for Protorthodes eureka

XML Treatment for Protorthodes incincta

XML Treatment for Protorthodes argentoppida

XML Treatment for Protorthodes mulina

XML Treatment for Protorthodes oviduca

XML Treatment for Protorthodes orobia

XML Treatment for Protorthodes melanopis

XML Treatment for Protorthodes texicana

XML Treatment for Protorthodes mexicana

XML Treatment for Protorthodes perforata

XML Treatment for Protorthodes rufula

XML Treatment for Protorthodes ustulata

XML Treatment for Protorthodes alfkenii

XML Treatment for Protorthodes antennata

XML Treatment for Nudorthodes

XML Treatment for Nudorthodes texana

XML Treatment for Nudorthodes variabilis

XML Treatment for Nudorthodes molino

## Check list of *Protorthodes* and *Nudorthodes*

**933103*** *Protorthodes curtica* (Smith, 1890)

syn. *Protorthodes bostura* (Smith, 1908)

**933104** *Protorthodes eureka* (Barnes & Benjamin, 1927)

**933105** *Protorthodes incincta* (Morrison, 1874)

syn. *Protorthodes utahensis* (Smith, [1888])

syn. *Protorthodes akalus* (Strecker, 1899)

syn. *Protorthodes saturnus* (Strecker, 1900)

syn. *Protorthodes smithii* (Dyar, 1904)

syn. *Protorthodes indra* (Smith, 1906)

syn. *Protorthodes coloradensis* (Strand, [1917])

syn. *Protorthodes daviesi* (Barnes & Benjamin, 1927)

**933107** *Protorthodes argentoppida* McDunnough, 1943

**933112** *Protorthodes mulina* (Schaus, 1894)

syn. *Protorthodes pseudochroma* (Dyar, 1913)

**933113** *Protorthodes oviduca* (Guenée, 1852)

syn. *Protorthodes capsella* (Grote, 1874)

syn. *Protorthodes lindrothi* Krogerus, 1954

**933114** *Protorthodes orobia* (Harvey, 1876)

**933115** *Protorthodes melanopis* (Hampson, 1905)

**933115.1** *Protorthodes texicana* Lafontaine, 2014

** *Protorthodes mexicana* Lafontaine, 2014

**933115.2** *Protorthodes perforata* (Grote, 1883)

syn. *Protorthodes constans* (Dyar, 1918)

**933115.3** *Protorthodes rufula* (Grote, 1874)

**933115.4** *Protorthodes ustulata* Lafontaine, Walsh & Ferris, 2014

**933115.5** *Protorthodes alfkenii* (Grote, 1895)

syn. *Perigea latens* (Smith, 1908)

syn. *Protorthodes occluna* (Smith, 1909)

**933115.6** *Protorthodes antennata* (Barnes & McDunnough, 1912)

**933116** *Nudorthodes texana* (Smith, 1900)

syn. *Nudorthodes consors* (Smith, 1900)

**933117** *Nudorthodes variabilis* (Barnes & McDunnough, 1912)

**933117.1** *Nudorthodes molino* Lafontaine, Walsh & Ferris, 2014

* Numbers adapted from Lafontaine & Schmidt (2010) check list

** Mexico only
